# Nortriptyline hydrochloride, a potential candidate for drug repurposing, inhibits gastric cancer by inducing oxidative stress by triggering the Keap1-Nrf2 pathway

**DOI:** 10.1038/s41598-024-56431-5

**Published:** 2024-03-13

**Authors:** Chunyang Zhu, Yangyang Lu, Shasha Wang, Jialin Song, Yixin Ding, Yan Wang, Chen Dong, Jiani Liu, Wensheng Qiu, Weiwei Qi

**Affiliations:** Department of Oncology, The Affiliated Hospital of Qingdao University, Qingdao, China

**Keywords:** Nortriptyline hydrochloride, Gastric cancer, Oxidative stress, ROS, Drug repurposing, Drug development, Cell death, Cell growth, Cell migration, Cell signalling, Gastric cancer

## Abstract

Effective drugs for the treatment of gastric cancer (GC) are still lacking. Nortriptyline Hydrochloride (NTP), a commonly used antidepressant medication, has been demonstrated by numerous studies to have antitumor effects. This study first validated the ability of NTP to inhibit GC and preliminarily explored its underlying mechanism. To begin with, NTP inhibits the activity of AGS and HGC27 cells (Human-derived GC cells) in a dose-dependent manner, as well as proliferation, cell cycle, and migration. Moreover, NTP induces cell apoptosis by upregulating BAX, BAD, and c-PARP and downregulating PARP and Bcl-2 expression. Furthermore, the mechanism of cell death caused by NTP is closely related to oxidative stress. NTP increases intracellular reactive oxygen species (ROS) and malondialdehyde (MDA) levels, decreasing the mitochondrial membrane potential (MMP) and inducing glucose (GSH) consumption. While the death of GC cells can be partially rescued by ROS inhibitor N-acetylcysteine (NAC). Mechanistically, NTP activates the Kelch-like ECH-associated protein (Keap1)—NF-E2-related factor 2 (Nrf2) pathway, which is an important pathway involved in oxidative stress. RNA sequencing and proteomics analysis further revealed molecular changes at the mRNA and protein levels and provided potential targets and pathways through differential gene expression analysis. In addition, NTP can inhibited tumor growth in nude mouse subcutaneous tumor models constructed respectively using AGS and MFC (mouse-derived GC cells), providing preliminary evidence of its effectiveness in vivo. In conclusion, our study demonstrated that NTP exhibits significant anti-GC activity and is anticipated to be a candidate for drug repurposing.

## Introduction

Gastric cancer (GC) has become the fifth most prevalent malignant tumor and the third most common cause of cancer-related death worldwide^1,2^. Although GC treatment has advanced, there is still a lack of effective drugs for treating GC, and the prognosis for GC patients is still poor. Drug resistance and dose-limiting toxicity are crucial issues limiting the efficacy of GC chemotherapeutics^3,4^. Therefore, seeking novel antitumor medications will be an important strategy for improving the prognosis and long-term survival rate of GC patients.

Psychological factors are significantly related to the survival and prognosis of cancer patients^5^. Patients who have depressive symptoms have shorter survival times and greater risks of mortality^6^. Nortriptyline hydrochloride (NTP) is one of the most frequently utilized clinical antidepressants. Many studies of NTP have shown that it has significant antitumor effects, yet the underlying mechanism is different in various malignancies. For example, NTP induces mitochondrial damage and FAS-FasL receptor-mediated apoptosis in bladder cancer cells^7^. NTP promoted cell death through lysosomal destruction in pineal blastomas^8^. By inhibiting macropinocytosis, NTP suppresses the fatty acid uptake of tumor cells, thereby inhibiting tumor growth^9^. It has also been shown to have powerful anticancer effects on colon cancer^10^, melanoma^11^ and multiple myeloma^12,13^. However, it is still unknown whether the anticancer effects of NTP are also effective in treating GC.

Oxidative stress (OS) is defined as an imbalance dominated by peroxide and is primarily manifested as the excessive generation of oxidative compounds such as ROS^14^. The abundant generated ROS can induce cell membrane rupture and mitochondrial dysfunction, consequently leading to cell death^15^. The Keap1-Nrf2 signaling pathway, one of the typical regulatory pathways connected with OS, has been found in various tumors. When there is a low concentration of ROS in tumor cells, Keap1 can bind to Nrf2 and promote Nrf2 degradation via the proteasome. Elevated OS and increased ROS levels can suppress the binding of Keap1 to Nrf2 and liberate Nrf2 for nuclear translocation, triggering and activating the transcription of a large number of antioxidant genes, including glutamic acid cysteine ligase modifier (GCLM), NAD(P)H quinone dehydrogenase 1 (NQO1) and heme oxygenase 1 (HO-1)^16^. Clinical antitumor therapies such as radiation and chemotherapy are currently effective by inducing OS^17,18^. In general, treatments that promote oxidation have shown superior results compared to antioxidation therapies in cancer treatment^19^.

In this study, we investigated the role of NTP in GC and further validated the underlying mechanisms involved. According to our research, NTP causes GC cell death and inhibits the proliferation and migration of GC cells. Further research demonstrated that NTP can induce cell apoptosis, oxidative stress, and antitumor activity in vivo. These results show the efficacy of NTP in the treatment of GC, and NTP has the potential to be used to convert antidepressants to GC therapy.

## Results

### NTP exerts anticancer activities in vitro

**Figure 1:**
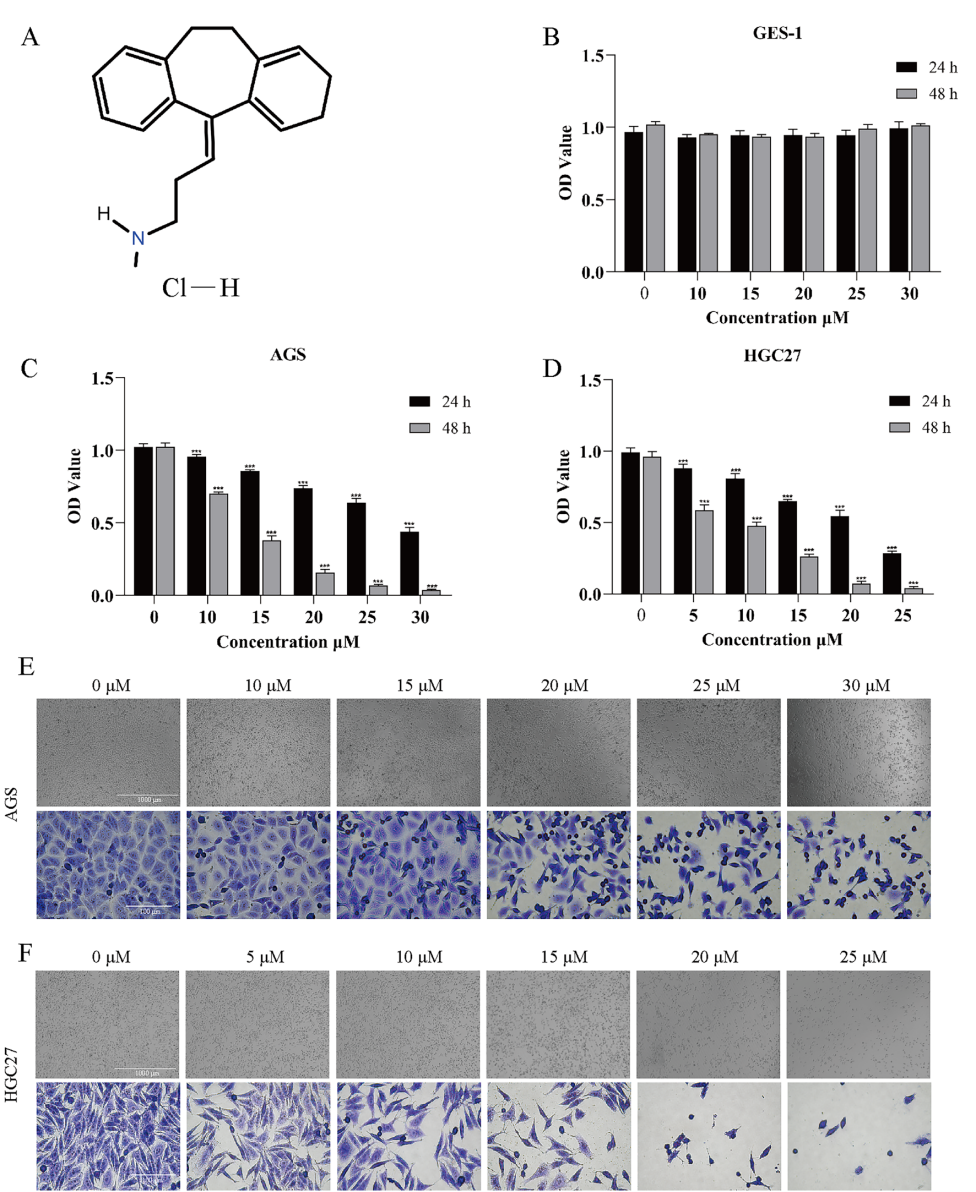
NTP exerts anticancer activities in GC cells in vitro. (**A**) The molecular structure of NTP. (**B**–**D**) AGS, HGC27, and GES1 cells were treated with different concentrations of NTP for 24 and 48 h, and their cell viability was measured using the MTT assay. (**E**,**F**) After 24 h of treatment with different concentrations of NTP, the density and morphology of AGS and HGC27 cells stained with crystal violet were observed via microscopy. Data are shown as the mean ± SD, with significant differences from the control group marked by *p < 0.05, **p < 0.01, ***p < 0.001.

In this investigation, the structure of NTP (MW, 299.84) used on cells and animals (Fig. 1A). Initially, we investigated whether NTP has an inhibitory effect on GC cells by evaluating its cytotoxicity in AGS, SGC7901, MKN7, HGC27, and NCI-N87 cells. Based on the pre-experimental results of MTT and relevant reference literature^20^, we selected two commonly used GC cell lines with different degrees of differentiation for parallel validation experiments based on the effectiveness of NTP in different cell lines. One type of cell is undifferentiated HGC27 cells, and the other type is adenocarcinoma AGS cells. Following preliminary experimental results, the concentrations of NTP used for GES1 and AGS cells were 0, 10, 15, 20, 25, and 30 μM, whereas those used for HGC27 cells were 0, 5, 10, 15, 20, and 25 μM. MTT assays revealed no significant cytotoxic effects at concentrations ranging from 0–30 µM for 24 h or 48 h in GES1 cells, indicating that GES1 cells could be safely accepted at this concentration range (Fig. 1B). At lower doses, NTP significantly inhibited the activity of HGC27 and AGS cells in a time- and concentration-dependent manner. After 24 h of NTP treatment, the IC50 of NTP in AGS cells was approximately 30 μM, while that in HGC27 cells was approximately 20 μM (Fig. 1C,D). MTT assays were also performed to determine the ability of NTP to inhibit MKN7, NCI-N87, and SGC7901 cell viability (Fig. S1A–C). Optical microscopy was used to evaluate alterations in cell quantity and morphology (split into two types with or without crystal violet staining). The number of cells decreased as the NTP concentration increased. Furthermore, the morphology of the cells degenerated and turned from the original appearance to wrinkled, amorphous, mutilated, and twisted shapes at high concentrations of NTP (Fig. 1E,F).

### NTP inhibits the proliferation and migration of GC cells

**Figure 2:**
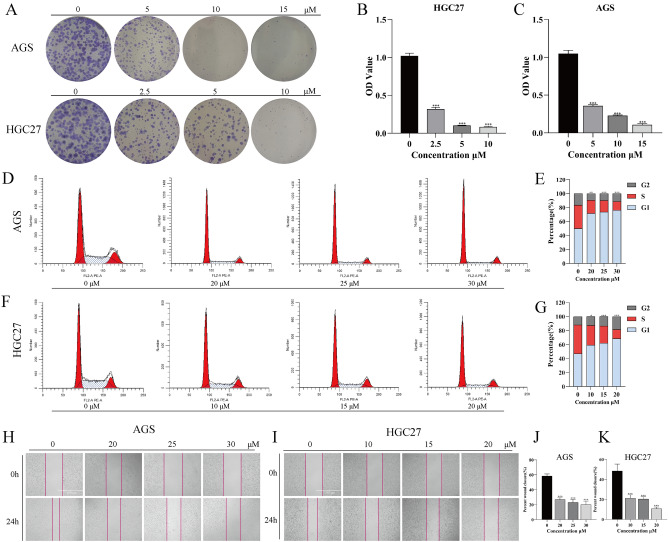
NTP inhibits the proliferation and migration of GC cells. (**A**) Typical colony formation assays were performed on AGS and HGC27 cells after 14 days of NTP treatment. (**B**,**C**) Quantitative analysis of the information obtained at 490 nm following the dissolution of AGS and HGC27 clones in glacial acetic acid. (**D**–**G**) The percentages of AGS and HGC27 cells in the G1, S, and G2 cell cycle phases. (**H**–**I**) The outcomes of the migration assay used on AGS and HGC27 cells that were NTP-treated at different times during the length of a 24 h period. (**J**,**K**) Quantitative analysis of the scratch migration rate was performed with ImageJ software. The data are shown as the mean ± SD, with significant differences from the control group marked by *p < 0.05, **p < 0.01, ***p < 0.001.

After confirming that NTP has a significant inhibitory effect on GC cells, we investigated whether NTP affects GC cell proliferation and migration. To investigate the effect of NTP on HGC27 and AGS cell proliferation, colony formation assays were performed. The results indicated that, compared with the control treatment, treatment of HGC27 and AGS cells with a low dose of NTP markedly inhibited tumor cell proliferation, and this effect was positively correlated with the NTP concentration (Fig. 2A). Then, the absorbance was measured after dissolving the cell colonies in 30% glacial acetic acid solution (Fig. 2B,C). The alterations in the cell cycle after 24 h of NTP therapy were then evaluated using flow cytometry. Compared to those in the control group, the proportions of HGC27 (20 µM) and AGS (30 µM) cells in the S phase were reduced by 27.98% and 22.28%, respectively, and the progressive shortening of the S phase was accompanied by an increase in the proportion of cells in the G1 phase (Fig. 2D–G). Cancer cell migration is closely linked to tumor metastasis. Migration and metastasis are linked to poor prognosis and play significant roles in the development of cancer, distant metastasis, and treatment resistance^21^. Wound healing assays were performed to evaluate the effect of NTP on GC cell migration. NTP significantly inhibited the migration of HGC27 and AGS cells (Fig. 2H,K). According to the above results, NTP could successfully inhibit HGC27 and AGS cell proliferation and migration.

### NTP destroys the cell membrane and induces apoptosis in GC cells

**Figure 3:**
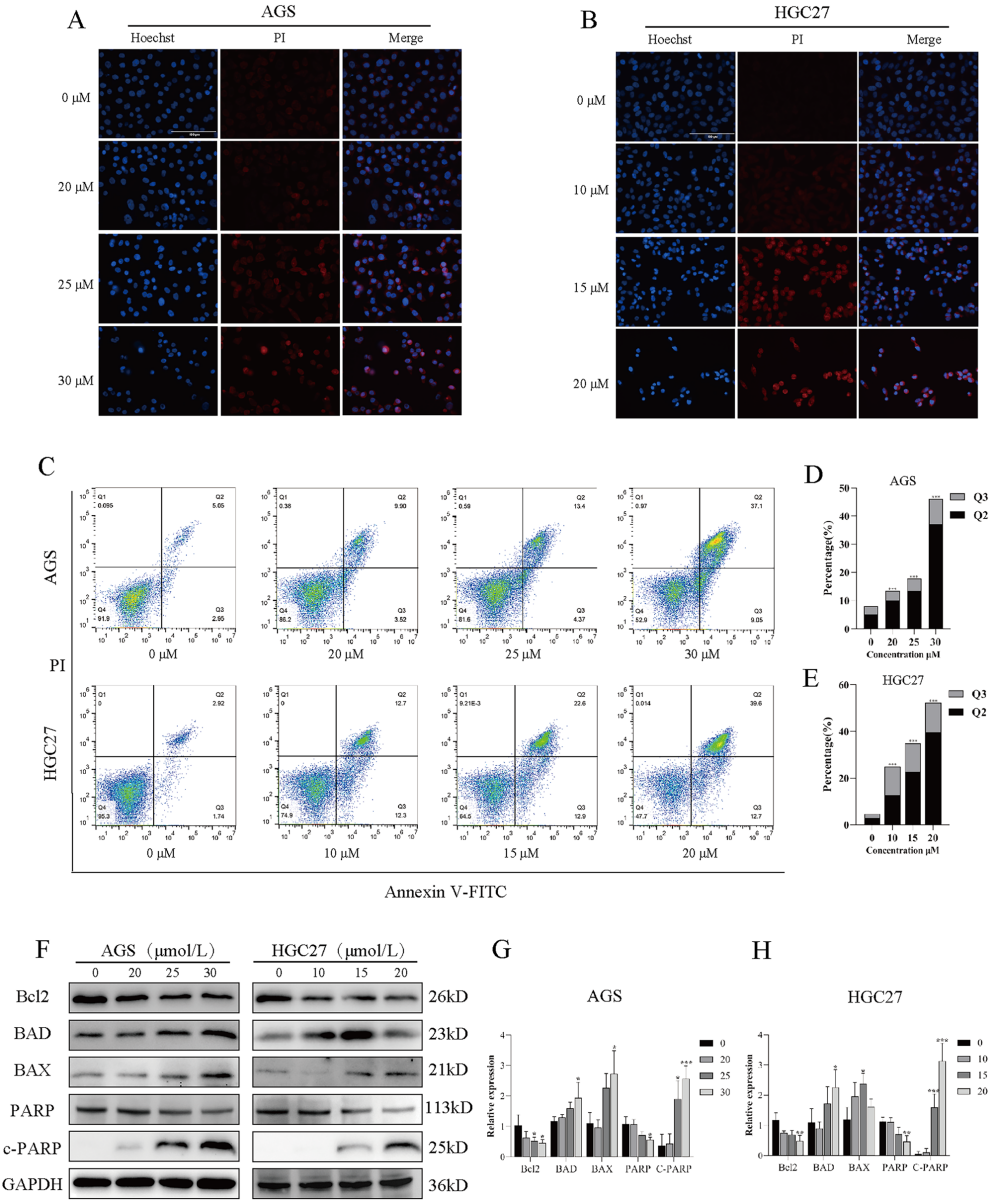
NTP destroys the cell membrane and induces apoptosis in GC cells. (**A**,**B**) Fluorescence images of AGS and HGC27 cells after 24 h of NTP treatment stained with Hoechst 33342/PI, with blue representing the nucleus and red representing the PI infiltrating the cells. (**C**–**E**) AGS and HGC27 cells underwent apoptosis after treatment with NTP for 24 h, as did the percentages of cells undergoing early and late apoptosis. (**F**–**H**) Western blotting was used to measure the expression of the apoptotic proteins Bcl2, BAX, BDA, PARP and C-PARP in AGS and HGC27 cells treated with NTP for 24 h. GAPDH levels were used in a quantitative measurement of protein expression levels. Data are shown as the mean ± SD, with significant differences from the control group marked by *p < 0.05, **p < 0.01, ***p < 0.001.

Next, we determined whether NTP damages the cell membrane and induces apoptosis. Hoechst/PI staining was performed after NTP treatment for 24 h in HGC27 and AGS cells at different concentrations. When the NTP concentration increased, an increasing amount of PI entered the cells, and the red fluorescence gradually increased (Fig. 3A,B). Flow cytometry was utilized to assess the rate of apoptosis in HGC27 and AGS cells after 24 h of NTP treatment. The overall apoptotic rates of HGC27 cells after NTP treatment were 4.66%, 25%, 34.9%, and 52.3% in the control, 10 μM, 15 μM, and 20 μM groups. The overall apoptotic rates of AGS cells after NTP treatment were 8%, 13.42%, 17.77%, and 46.15% in the control, 20 μM, 25 μM, and 30 μM groups. (Fig. 3C–E). After 24 h of NTP treatment, there were changes in the expression of apoptosis-related proteins in HGC27 and AGS cells compared to those in the control group. Western blotting confirmed these findings; the expression of BAX, BAD, and c-PARP increased, while that of Bcl2 and PARP decreased (Fig. 3F–H). The above results suggest that NTP can induce apoptosis in GC cells.

### NTP induces oxidative stress in GC cells

**Figure 4:**
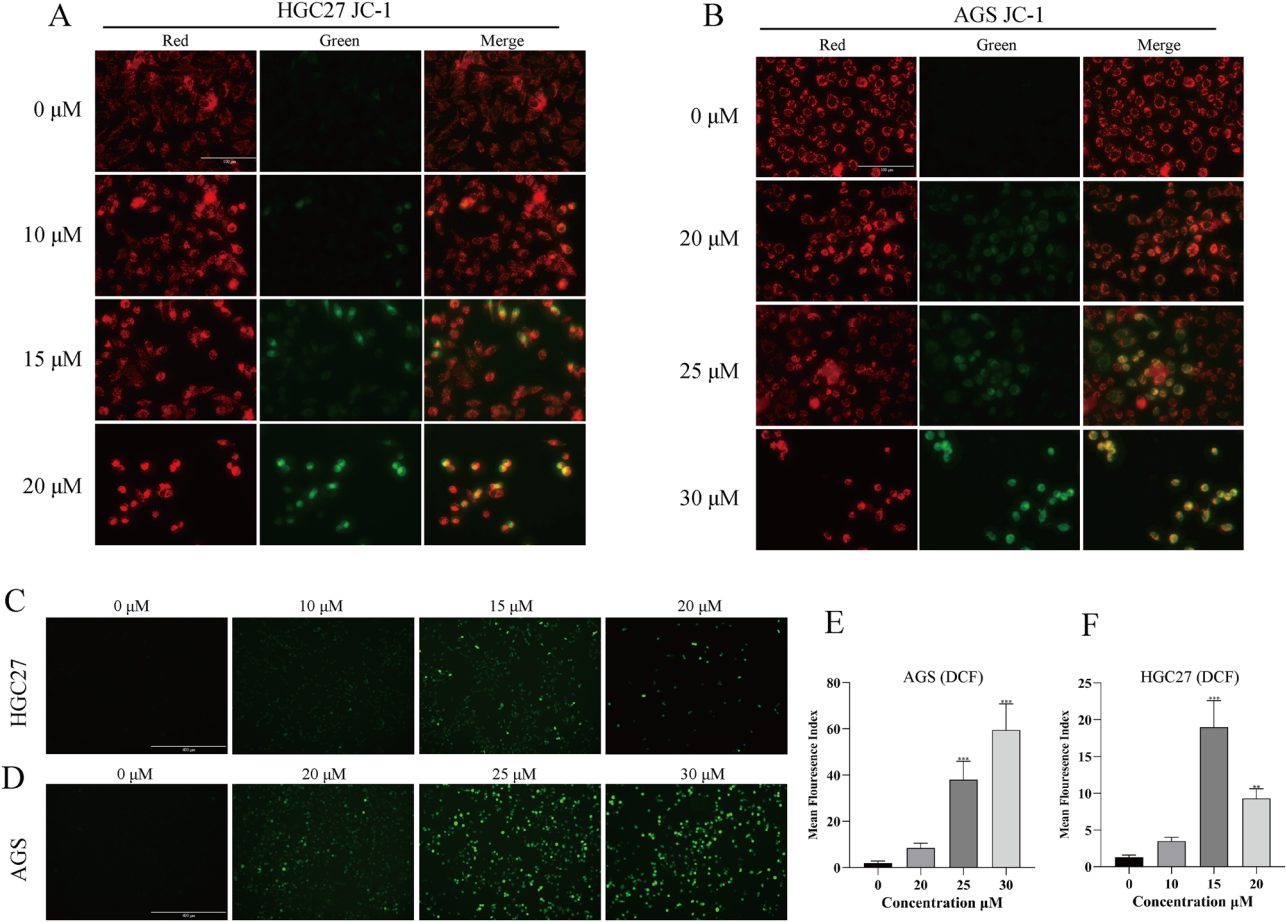
NTP induces oxidative stress in GC cells. (**A**,**B**) Fluorescence staining of JC-1 in AGS and HGC27 cells after 24 h of NTP treatment showed red fluorescence representing JC-1 aggregates and green fluorescence representing JC-1 monomers caused by damaged mitochondria. (**C**–**F**) Following 24 h of NTP treatment, the ROS in AGS and HGC27 cells were stained with DCF, and ImageJ was used to assess the green fluorescence intensity. Data are shown as the mean ± SD, with significant differences from the control group marked by *p < 0.05, **p < 0.01, ***p < 0.001.

Based on the above experimental results, we further explored how NTP leads to cell death. The decrease in mitochondrial membrane potential (MMP) and the excessive production of ROS are important indicators of oxidative stress. When mitochondrial activity remains unaffected, JC-1 collects in the mitochondrial matrix, forming JC-1 polymers that are light red. On the other hand, when the MMP is compromised, JC-1 monomers that are unable to assemble in the mitochondrial matrix glow green. The results demonstrated that following staining with JC-1, the intensity of the green fluorescence in HGC27 and AGS cells gradually increased (Fig. 4A,B). It was evident that NTP can decrease the MMP and harm mitochondrial activity. DCF was used to investigate whether ROS increase in GC cells treated with NTP for 24 h, as numerous studies have demonstrated that the induction of excessive ROS is an anticancer target in malignancies. The formation of ROS (green fluorescence), which was strongly increased in HGC27 and AGS cells following NTP treatment, was demonstrated by fluorescence data and was positively correlated with the NTP concentration (Fig. 4C–F).

### NTP disrupts redox homeostasis and activates the Keap1-Nrf2 pathway

**Figure 5:**
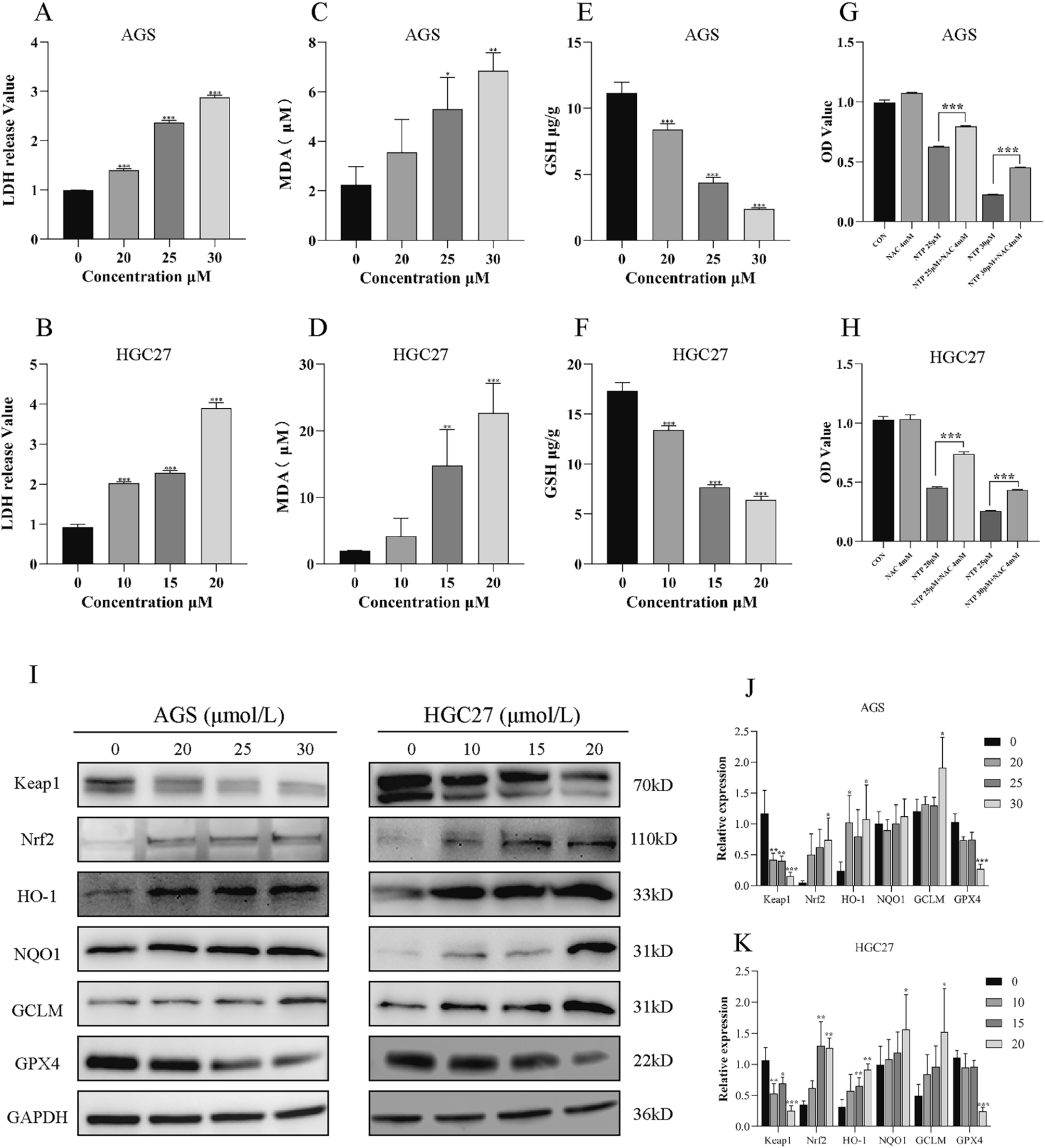
NTP disrupts redox homeostasis and activates the Keap1-Nrf2 pathway. (**A**–**F**) LDH, MDA, and GSH levels were measured in AGS and HGC27 cells following 24 h of NTP treatment. (**G**–**H**) After 24 h, AGS and HGC27 cells were cocultured in the presence or absence of 4 mM NAC, and MTT was used to assess the viability of the cells. (**I**–**K**) In AGS and HGC27 cells treated with NTP for 24 h, the Keap1, Nrf2, HO-1, NQO1, GCLM, and GPX4 proteins associated with oxidative stress were identified via Western blotting. GAPDH levels were used in a quantitative measurement of protein expression levels. Data are shown as the mean ± SD, with significant differences from the control group marked by *p < 0.05, **p < 0.01, ***p < 0.001.

The LDH from the cytoplasm was incremented with increased NTP concentration (Fig. 5A,B). Consequently, we examined the oxidative and reducing chemicals involved in OS. MDA is an indicator of ROS-induced lipid peroxidation damage, while GSH is the most essential thiol antioxidant in cells and has important antioxidant capabilities. After 24 h of NTP treatment, HGC27 and AGS cells produced more MDA while consuming more GSH than did the control cells (Fig. 5C–F). In addition, HGC27 and AGS cells were cocultured with NTP and NAC (a ROS inhibitor) for 24 h to determine whether NTP-induced cell death is connected to the generation of ROS. MTT assays revealed a decrease in the number of dead cells in the NAC + NTP group compared to the same concentration in the NTP group, showing that ROS are involved in some of the cytotoxicity caused by NTP (Fig. 5G–H). Under OS, Keap1 dissociates from Nrf2 and enhances Nrf2 nuclear translocation, improving the expression of the Nrf2 downstream genes HO-1, GCLM and NQO1^16^. The expression of Keap1-Nrf2 pathway components was assessed by western blotting to verify protein expression after 24 h of NTP treatment. Compared with that in the control group, the expression of Keap1 in the NTP treatment group decreased, while the expression of Nrf2, HO-1, GCLM, and NQO1 increased. Furthermore, we found that the expression of GPX4, which can catalyze the creation of the reducing substance GSH to combat ROS, decreased after NTP treatment (Fig. 5I–K). In summary, NTP induced OS in GC cells, causing damage to the cell membrane and mitochondrial activities, resulting in increased levels of oxidizing substances, decreased levels of reducing substances in cells, and activation of the Keap1-Nrf2 signaling pathway.

### Preliminary differential gene expression and pathway enrichment through RNA sequencing

**Figure 6:**
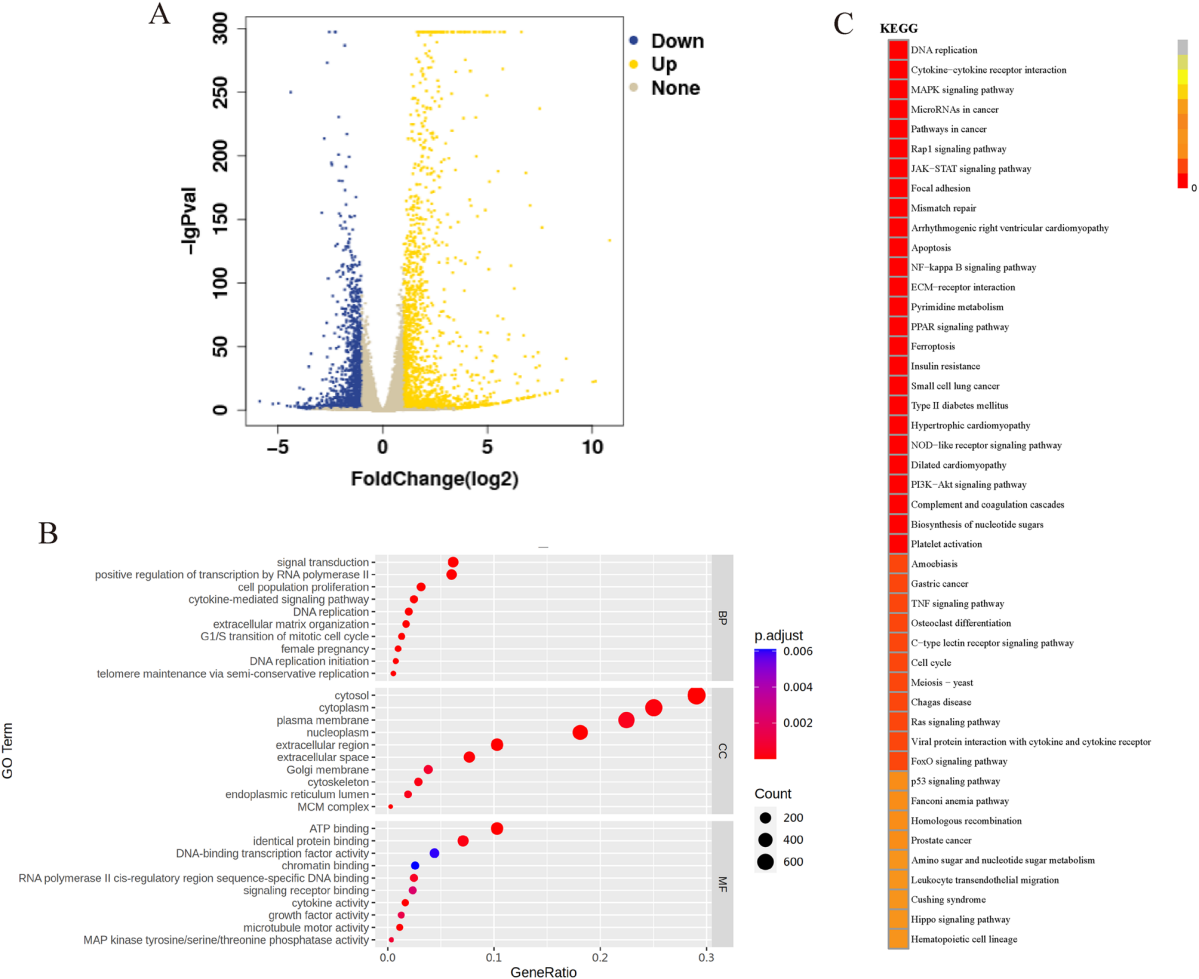
Preliminary differential gene expression and pathway enrichment through RNA sequencing. (**A**) In AGS cells, the gene expression between the NTP-treated 24 h group and the control group differed significantly, as shown by the volcano map. A multiple of differences higher than or equal to 2 and a q value less than 0.05 are the screening standards for differentially expressed genes. (**B**,**C**) GO and KEGG analyses of the genes that were significantly altered following NTP treatment.

The following was conducted to investigate the molecular mechanism by which NTP inhibits GC. Total RNA was collected from the control group of AGS cells and from the group treated with NTP (30 µM) for 24 h. The RNA sequencing data revealed 3302 differentially expressed genes (DEGs), comprising 1345 downregulated and 1957 upregulated genes, in the treatment group compared to the control group (Fig. 6A). The effects of the DEGs on GC cells were investigated using Gene Ontology (GO) and Kyoto Encyclopedia of Genes and Genomes (KEGG) analyses. The biological process (BP) category was enriched mainly in cell population proliferation, DNA replication, and the G1/S transition of the mitotic cell cycle. The cellular component (CC) and molecular function (MF) categories were enriched mainly in the MCM complex, ATP binding, and growth factor activity (Fig. 6B). In gastrointestinal cancer, the MCM gene family refers to a gene family that encodes proteins that play critical roles in cell mitosis and DNA replication^22^. Cluster heatmaps were created using transcriptome differential gene data for the MCM family and the proliferation-promoting genes CDC6 and CDC45. The results showed a decrease in the expression of these genes during NTP treatment, suggesting that NTP may inhibit GC proliferation (Fig. S2). GO analyses suggested that the effects of these drugs on GC cells may include the inhibition of cell proliferation, interference with DNA replication and cell cycle regulation, and interference with intracellular energy metabolism and growth factor activity. These effects may help inhibit the development and growth of GC. According to the KEGG analysis, these DEGs were substantially enriched in the apoptosis, ferroptosis and multiple signaling pathways, including the PI3K-Akt, MAPK, and JAK-STAT pathways (Fig. 6C). In summary, NTP may inhibit the progression of GC by promoting apoptosis, inducing ferroptosis, and regulating multiple important signaling pathways.

### Proteomic analysis further confirms the mechanism of action of NTP

**Figure 7:**
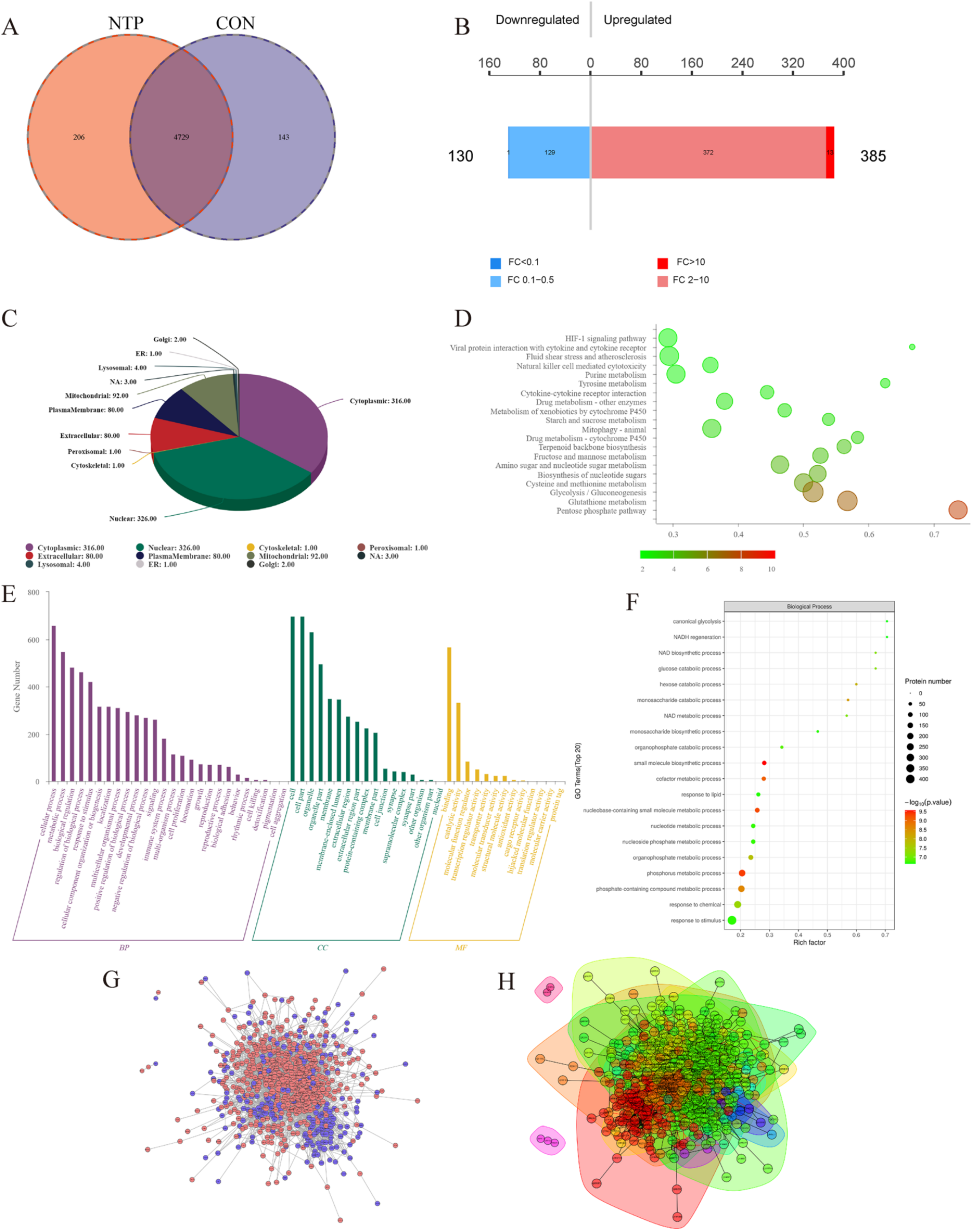
Proteomic analysis further explored the effects of NTP on GC cells. (**A**) Protein overlap between the 24 h NTP treatment group and the control group was determined via Wayne plot analysis. (**B**) Analysis of the proteins with expression differences between groups with a fold change (FC) > 2 and P value less than 0.05 to determine how many up- and downregulated proteins are present in the comparison groups. (**C**) Subcellular localization of the differentially expressed proteins. (**D**,**E**) KEGG analysis and GO analysis of the significantly differentially expressed proteins. (**F**) Using Fisher's exact test, the GO function enrichment bubble chart was generated for the biological process classification. (**G**,**H**) Protein clustering and protein interaction network analyses were separated into many clusters in the interaction network diagram.

We next performed proteomic analysis on AGS cells treated with NTP (30 µM) for 24 h according to previous transcriptional level research. The results revealed that 4729 gene crossings were annotated between the treatment and control groups. Further analysis of the NTP group revealed 129 downregulated genes and 372 upregulated genes compared to those in the control group (Fig. 7A,B). In each organelle, various proteins often have diverse biological activities. Analysis of the subcellular localization of the DEGs after NTP treatment revealed that 92 proteins were concentrated in the mitochondria (Fig. 7C). Mitochondria are important organelles within cells and are involved in key processes, such as energy metabolism, the respiratory chain, oxidative phosphorylation, and the regulation of cell apoptosis. These results suggest that the action of NTP on the mitochondria of GC cells may have an impact on cell energy metabolism, oxidative stress and antioxidant activity, as well as on cell apoptosis regulation. GO and KEGG enrichment analyses were performed on all the differentially expressed proteins, and the results revealed that these proteins were enriched mainly in metabolic processes, biological control processes, and stimuli-related stress regulation (Fig. 7D,E). A protein network interaction map was constructed based on the interactions between the differentially expressed proteins in the NTP group, and the highly aggregated proteins were separated into clusters (Fig. 7G,H).

### NTP suppresses subcutaneous tumor growth in vivo

**Figure 8:**
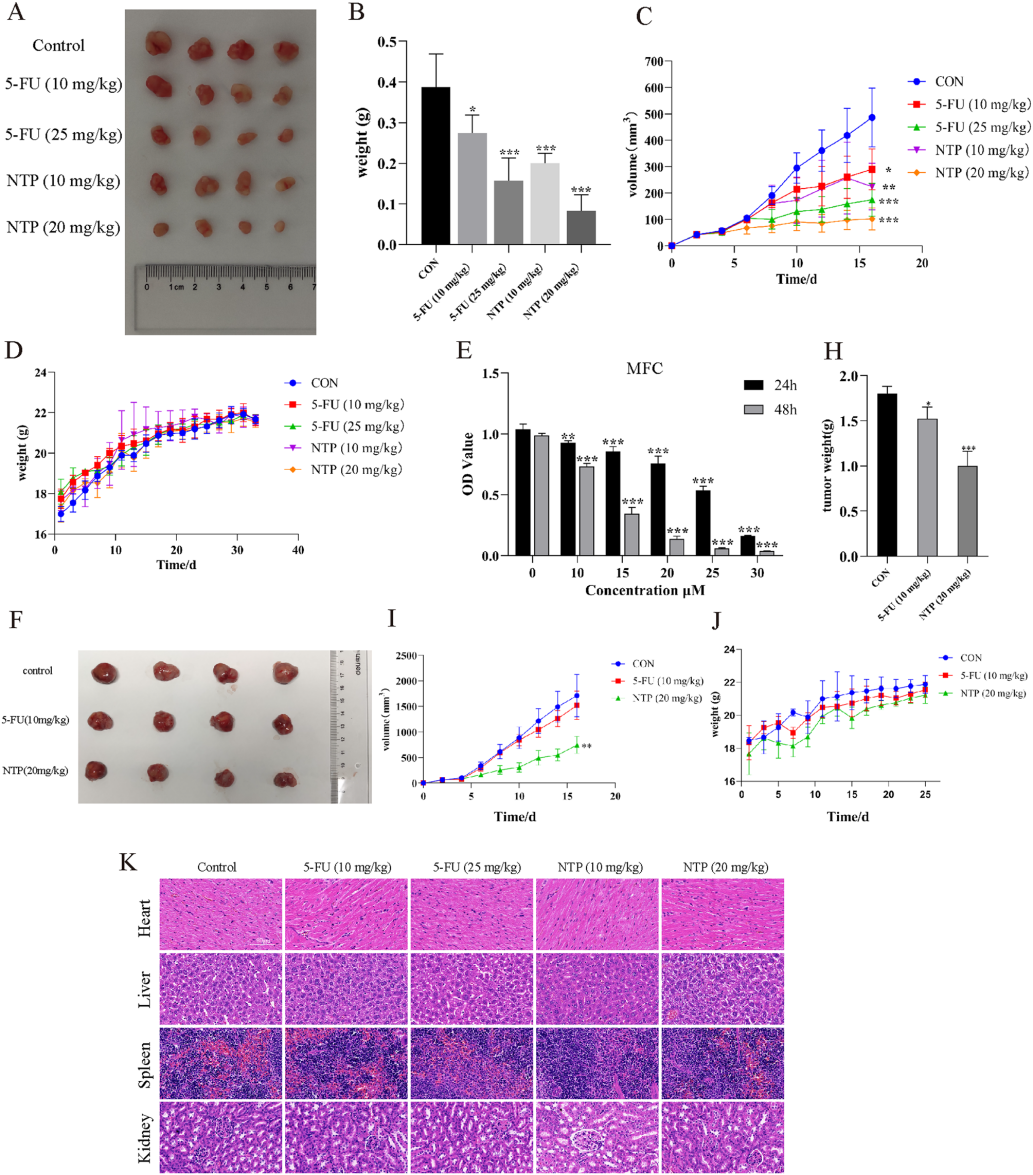
NTP suppresses subcutaneous tumor growth in vivo. (**A**) Images of tumors (generated by subcutaneous tumorigenesis of AGS cells) from the five groups of nude mice at the end of the treatment period. (**B**,**C**) Statistical analysis of the tumor weights and sizes. (**D**) Changes in the weights of the nude mice from the time of purchase to the time of euthanasia. (**E**) MTT was used to determine the viability of MFC. (**F**) Images of subcutaneous tumors in the three groups (subcutaneous tumorigenesis of MFC cells). (**H**–**J**) The volume and weight of tumors and a record of the weight of the mice. (**K**) Important organs stained with HE. Data are shown as the mean ± SD, with significant differences from the control group marked by *p < 0.05, **p < 0.01, ***p < 0.001.

The nude mouse subcutaneous tumor model was established to determine whether NTP also has antitumor effects in vivo. The nude mice were subcutaneously injected with 5 × 10^6^ AGS cells into the subcutaneous region located in the right axilla. When the tumor volume was between 40 and 50 mm^3^, the mice were randomly assigned five groups, 4 mice in each group: the negative control group (DMSO and solvent), positive control group (5-FU, 10 mg/kg or 25 mg/kg), and treatment group (NTP, 10 mg/kg or 20 mg/kg). All the mice were intraperitoneally injected every two days. The concentrations of NTP and 5-FU used in this study, as well as the frequency of drug injection, were referenced from relevant literature^7,9,23,24^. The mice were terminated by humanitarian intervention after 16 days of treatment. The tumors were harvested and photographed after drug treatment (Fig. 8A). Compared to those in the control group, both 5-FU- and NTP-treated tumors exhibited smaller tumor volumes and weights (Fig. 8B,C). The body weight curves of the five groups showed no significant differences, indicating the tolerability of the drugs (Fig. 8D). Mouse-derived MFC cells were also used for subcutaneous tumor formation model validation. Mouse-derived MFC cells were chosen to produce subcutaneous tumors in nude mice. The in vitro assay demonstrated that NTP treatment had the similar impact on cell viability after 24 h and 48 h (Fig. 8E). The nude mice were subcutaneously injected with 2.5 × 10^5^ MFC cells into the subcutaneous region located on the right hind limb. When the tumor volume was between 50 and 80 mm^3^, the mice were randomly assigned three groups, 4 mice in each group: the negative control group (DMSO and solvent), positive control group (5-Fu, 10 mg/kg), and treatment group (NTP, 20 mg/kg). All the mice were intraperitoneally injected every two days. The mice were terminated by humanitarian intervention after 14 days of treatment, and the results showed that the tumor volume and weight in the NTP and 5-FU treatment groups were lower than those in the negative control group (Fig. 8F–I). The body weight curves of the three groups also showed no significant differences (Fig. 8J). We performed a blood cell analyzer test on nude mouse blood samples, as well as H&E staining of vital organs, to determine whether NTP is dangerous to hosts. The results of blood cell analyzer test (Supplementary Table S1) and H&E staining showed no significant difference between the control group and treatment group (Fig. 8K), indicating that the effective therapeutic concentration of NTP did not cause significant harmful or side effects during the in vivo treatment process.

## Discussion

Currently, fluorouracil or platinum medicines combined with paclitaxel are still the most frequently utilized treatments for advanced GC, but the efficacy is not sufficient^25^. Although targeted therapy and immunotherapy have shown impressive efficacy and extended survival in patients, a large number of patients still cannot benefit^26,27^. It is critical to develop efficient anti-GC medicines for advanced-stage patients.

Numerous studies have demonstrated that tricyclic antidepressants such as imipramine, amitriptyline, and clomipramine have significant antitumor effects^28^. The existing treatment methods and drug discovery methods are being used in a novel way, namely, drug repurposing. Compared to the development of new drugs, this approach will likely produce more rapid clinical results at a lower cost. To propose new prospective therapeutic strategies for GC, this study examined the function of NTP (a conventional TCA) in the treatment of GC.

First, through MTT, colony formation, cell cycle flow cytometry, and wound healing assays, we demonstrated that NTP at relatively low concentrations has significant cytotoxic and inhibitory effects on GC. Next, we focused on the most common apoptotic phenomenon associated with cancer mortality^29^. Hoechst fluorescence staining, apoptotic flow cytometry data, and apoptotic protein levels confirmed that NTP induced apoptosis in GC cells. Fluorescence data revealed dense and concentrated fluorescence in the nucleus, while flow cytometry data revealed a gradual increase in the number of early and late apoptotic cells, which was positively correlated with the NTP concentration. The upregulation of the proapoptotic proteins Bax, Bad, and c-PARP, as well as the downregulation of the antiapoptotic protein Bcl-2, were also observed.

However, further exploration is needed to determine the ways in which NTP produces cytotoxic effects. Targeted induction of oxidative stress, which directly generates an enormous amount of ROS or damages cancer cells' antioxidant systems, has shown significant antitumor effects^30,31^. Excessive ROS can have fatal cytotoxic effects through a variety of mechanisms, including compromised mitochondrial function, which in turn activates endogenous apoptotic pathways and disrupts the integrity of the phospholipid bilayer of the cell membrane, which results in cell death^32,33^. ROS and mitochondrial JC-1 staining were performed on GC cells treated with NTP, and the results showed that intracellular ROS accumulated and that the MMP was damaged. As the concentration of NTP increased, the results of PI staining (gradually increasing red fluorescence) and increased LDH release indicated damage to the cell membrane. The alterations in redox components after NTP treatment are reflected in the increase in intracellular MDA content and the reduction in GSH content, further indicating that the disruption of redox homeostasis is dominated by OS. NTP has the same ability to induce OS as clinically approved drugs or other drugs being explored. Clinical chemotherapeutic medicines, for example, 5-fluorouracil, doxorubicin, and cisplatin, can cause significant production of ROS^34,35^. Small molecule compounds (elesclomol)^36^ and classical medications (ivermectin)^37,38^ have been shown to have significant pro-oxidative effects^39,40^. Moreover, the ROS inhibitor NAC partially reversed NTP-induced cell death. As a result, we believe that increased ROS levels are one of the causes of NTP-induced death in GC cells.

We explored the Keap1-Nrf2 pathway as a mechanism involved in OS processes. When exposed to OS, cysteine residues in Keap1 are changed, resulting in a conformational shift and blockage of the proteasome degradation process, allowing newly produced Nrf2 to reach the nucleus and control target gene expression (NQO1, GCLM, and HO-1)^41^. Western blotting confirmed the activation of the Keap1-Nrf2-NOQ1/GCLM signaling pathway in GC cells after NTP treatment. Targeting Nrf2 activation also seems to be an antitumor approach. As an Nrf2 activator, sulforaphane could improve the effects of radiation on prostate cancer^42^. Sarmentosin Activates Nrf2 in Hepatocellular Carcinoma to Induce Autophagy-Dependent Apoptosis^43^.

Further analysis via RNA sequencing and quantitative proteomics also confirmed the potential anti-GC mechanism of NTP. The enrichment analysis of the DEGs revealed cell proliferation, DNA replication, cell cycle regulation, intracellular metabolic processes and apoptosis, ferroptosis, and the PI3K-Akt, MAPK, and JAK-STAT pathways. These results preliminarily suggested that NTP inhibits the development and growth of GC and provided direction for further exploration of the antitumor mechanism of NTP. A limitation of this study is the underutilization of omics data, as the analysis of DEGs provides only a simplistic understanding of the processes in which NTP is involved. The findings from the GO and KEGG analyses did not provide a comprehensive representation of the antitumor potential of NTP. Therefore, further investigations and verification are necessary to elucidate the profound inhibitory effects of NTP on GC.

In this study, nude mouse subcutaneous tumor models were established using AGS and MFC cells, respectively. The results showed that NTP could inhibit the growth of tumors, and there was no obvious host toxicity according to the weight of the mice and HE staining of important organs. It is worth noting that the 5-FU (10 mg/kg) group as a positive control did not inhibit tumor growth from MFC sources. Referring to two studies using MFC for tumor formation and both treated with a dose of 5-FU (10 mg/kg), the first study used 5-FU (10 mg/kg, once a day), which significantly decreased tumor size compared to that in the control group^44^, while the second study used 5-FU (10 mg/kg, once every two days), which did not significantly change tumor size compared to that in the control group^45^. A comparison of the two studies revealed that the failure to inhibit tumor growth may be related to the frequency of drug injection, as our study used 5-FU (10 mg/kg, once every two days). This issue needs attention and improvement in future experiments. Additionally, it is important to acknowledge the limitations of the animal experiments in this study. First, there is a concern regarding the small sample size, as only four nude mice were included in each group (n = 4). Moreover, the study utilized solely male mice, which raises a potential sex bias. This study did not perform immunohistochemical or immunofluorescence detection of proteins, such as Ki67, apoptosis-related proteins, or oxidative stress-related proteins, in tumor tissues. Western blot analysis was also not performed on tumor tissue, leading to a gap in the mechanistic understanding of the in vivo experiments. In further research on the mechanism by which NTP inhibits GC, we will pay attention to and address these issues.

Finally, based on the results of this study, shortcomings and prospects are proposed. First, Keap1 downregulation and Nrf2 activation could only indicate that the cell is during OS condition. Many studies have demonstrated that Nrf2 suppression has significant anticancer effects^46–50^. In addition, research on disulfiram is worthwhile to explore, the level of Nrf2 increases after cells are treated with disulfiram, and the cytotoxic effect of disulfiram can be considerably enhanced by knocking down Nrf2^51^. However, whether inhibiting Nrf2 expression in GC cells can improve NTP efficacy needs to be explored. Second, it is unclear whether Nrf2 is an oncogene or a tumor suppressor gene. For example, a recent study revealed that overactivating Nrf2 could inhibit cancer cell growth by reducing stress. Overexpression of Nrf2 reduced the survival rate of non-small cell lung cancer cells by causing substantial NADH reductive stress^52^. Since the HGC27 and AGS cells used in this study were not Nrf2-dependent cancer cells, we found that genes related to NADH regeneration, NAD biosynthetic processes, and NAD metabolic processes were significantly enriched in these processes after further analysis of the biological processes (Fig. 7F). However, whether NTP-induced GC cell death and Nrf2 activation involve reducing stress remains to be verified.

## Conclusion

In conclusion, NTP exhibits significant antitumor activity both in vivo and in vitro and can inhibit gastric cancer by inducing apoptosis and oxidative stress. This study initially demonstrated that the tricyclic antidepressant nortriptyline hydrochloride has a therapeutic impact on gastric cancer, indicating the prospect of medication reuse in the treatment of gastric cancer.

## Materials and methods

### Cell culture and reagents

Human GC cells (HGC-27 and AGS), human gastric mucosa GES-1 cells, and mouse GC cells MFC were obtained from the Central Laboratory of the Affiliated Hospital of Qingdao University, China. HGC27 cells were cultured in Roswell Park Memorial Institute (RPMI)-1640 medium (HyClone, USA) containing 20% fetal bovine serum (FBS) (Life-ilab, China) and 100 U/mL penicillin (Life-ilab, China). AGS, MFC, and GES1 cell-specific culture media were obtained from Procell Life Science & Technology Co., Ltd. (Wuhan, China). The cells were incubated at a constant temperature of 37°C, 5% CO2, and adequate humidity.

Nortriptyline Hydrochloride (N136755) was purchased from Aladdin (Shanghai, China). NTP was prepared with dimethyl sulfoxide (DMSO) (Solarbio, China) at a concentration of 100 mM as the storage solution. Bad (A19595), Bax (A19684) and Cleaved PARP (A19612) were purchased from ABclonal Technology Co., Ltd. (Wuhan, China). Nrf2 (ab62352), Keap1 (ab227828), GCLM (ab126704), NQO1 (ab80588), HO-1 (ab189491), PARP (ab191217), Bcl2 (ab32124) and GPX4 (ab125066) were purchased from Abcam.

### Cell viability assay

3-(4,5-Dimethylthiazol-2-yl)-2,5-diphenyltetrazolium bromide (MTT, M158055), which was used to assess cell viability, was obtained from Aladdin. Briefly, 30,000 cells/pores were seeded in a 24-well plate with 4 compound pores per group. GES1, HGC27, AGS, and MFC cells were treated with DMSO and different concentrations of NTP for 24 h and 48 h. The original culture media was discarded when the processing time was complete, and then, 500 μL of FBS-free MTT working solution was incubated for 2 h. After incubation, the MTT working solution was discarded, and the cells were dissolved in DMSO and the absorbance was measured at 490 nm using a full-function microplate detector.

### Colony formation assays

A total of 1000 HGC27 and AGS cells per well were seeded into each well of a 6-well plate, which was in the logarithmic growth stage. After 72 h of cell growth, the original medium was discarded, and new medium was added with various concentrations of NTP. The medium was then replaced every three days. After 14 days of treatment, the cells were fixed with 4% paraformaldehyde at room temperature for 30 min before being stained with 0.1% crystal violet (C110703, Aladdin) at room temperature for 2 h.

### Wound healing assay

3*10^5^ HGC27 and AGS cells were added in a 6-well plate. 200 µL tips was used to draw a horizontal line when the cell confluence was approximately 90%. The cells were then washed twice with phosphate-buffered saline (PBS) to eliminate the floating cells. After wound induction, the cells were treated with the specified NTP concentration for 24 h and imaged under the microscope at 0 h and 24 h. ImageJ was used to calculate the wound healing rate by measuring the area size and normalizing that to the control group.

### Hoechst 33342/PI staining

Round coverslips were pre-placed in a 24-well plate, and the same cell density was added using the abovementioned MTT detection method. After 24 h of NTP treatment, the cells were washed twice with PBS and then fixed in 4% paraformaldehyde for 30 min. The fixed round coverslip was placed upside down on a slide containing Hoechst 33342/PI anti-fluorescence quenching sealing solution (P0137, Beyotime, China). Following the completion of the sealing, pictures were taken using a fluorescence microscope.

### Cell apoptosis and cell cycle assays

2.5 × 10^5^ HGC27 and AGS cells were seeded in a 6-well plate the next day and treated with NTP for 24 h. Trypsinization without EDTA was used for obtaining the cells, which were then rinsed with precooled PBS and centrifuged twice. The Annexin V-FITC/PI apoptosis assay kit (40302, Yeasen, China) was incubated for 15 min according to the manufacturer's instructions, and the apoptosis rate was measured using flow cytometry.

The plates were treated with NTP as mentioned above. After treatment, the cells were collected by trypsinization in 1.5 mL EP tubes, centrifuged and fixed with 75% ethanol overnight. The cells were stained with propyl iodide according to the Cell Cycle and Apoptosis Analysis Kit (C1052, Beyotime). The cell cycle was detected by flow cytometry.

### Intracellular ROS measuring

Intracellular ROS in 2′,7′-dichlorodihydrofluorescein diacetate (DCF, D6883, Sigma) staining. DMSO was used to configure the DCF powder as a 10 mM storage solution. HGC27 and AGS cells were treated with different concentrations of NTP in 6-well plates for 24 h, and the stock solution was diluted to 10 μM working liquor at a ratio of 1:1000 with basic medium, and 2 mL of working liquor was added to each well. The cells were incubated in a cell incubator for 30 min without light, and then switched to the green fluorescence channel under a fluorescence microscope to observe the fluorescence intensity and take photos.

### MDA assay

MDA content in cells was determined using a malondialdehyde content assay kit (BC0025, Solarbio). In a 10 cm petri plate, HGC27 and AGS cells were seeded. After 24 h of NTP treatment, 5 million cells from each group were collected, the supernatant was spun, and the absorbance of the samples at 532 nm was measured using full-function microplate detector. MDA levels were determined using absorbance.

### LDH release experiment

The toxicity of the medication to cells was determined using the LDH Cytotoxicity Assay Kit (C0016, Beyotime). After 24 h of treatment with various concentrations of NTP, the supernatant of HGC27 and AGS cells was collected in 6-well plates. 120 μL of the supernatant was then transferred to 96-well plates, where the configured LDH detection reagent was applied. The absorbance at 490 nm was measured using full-function microplate detector after the orifice plates had been incubated on a shaking table at room temperature for 30 min.

### Western blotting

Cell lysis buffer for Western and IP (P0013, Beyotime) was used to lyse HGC27 and AGS cells after they had been exposed to various doses of NTP for 24 h. The BCA protein Colorimetric Assay Kit (E-BC-K318-M, Elabscience) was subsequently utilized to determine the total protein. After the protein was separated by electrophoresis, the membrane was transferred using polyvinylidene difluoride (PVDF) at an ice bath temperature. Five percent skim milk was used to block the portions. In a 4 °C shaker, the matching antibodies were incubated overnight. After rinsing with Tris-buffered saline and Tween (TBST), the film was treated with secondary antibodies (S0002, Affinity) for 1.5 h at room temperature. Finally, the imaging system was used to build the improved ECL chemiluminescent substrate kit (36222, Yeasen). For some of the PVDF membranes, Western blot fast stripping buffer (PS107, Epizyme) was used. After completing Western blot luminescence detection, the PVDF membranes were rinsed with TBST for 5 min. Add 10 mL of fast stripping buffer to cover the membrane and rinse for 20 min. The stripping solution was removed, and TBST was added to rinse the PVDF membrane for 5 min. The membrane was sealed with skim milk powder for 2 h, the PVDF membrane was washed with TBST, and then the other antibodies were added again. The gray value was analyzed and calculated using ImageJ software.

### RNA sequencing and proteomics analysis

AGC cells were treated in 30 µM concentration of NTP for 24 h. TRIzol Reagent (Seville Biotechnology, Wuhan) was added to extract total RNA from cell samples. The samples were submitted to Agilent Yoda Gene Technology Co., Ltd. (Beijing, China) for subsequent mRNA library construction and computer sequencing. The analysis process is mainly divided into three parts: sequencing data quality control, data comparison analysis and deep transcriptome analysis.

AGC cells were treated in 30 µM concentration of NTP for 24 h. After the treatment, the original culture medium was discarded, the cells were washed twice with PBS, and the cells were gently collected with a cell scraper in 1.5 mL EP tubes. The cells were centrifuged at 3000 rpm/min for 5 min and placed in a − 80° refrigerator. The collected samples are used for constructing and sequencing a proteomic quantitative library. The analysis process mainly includes two stages: mass spectrometry experiments and data analysis. The process of mass spectrometry analysis mainly includes protein extraction, peptide enzymolysis, chromatographic classification, liquid chromatography-tandem mass spectrometry (LC–MS/MS) data collection and database retrieval. Then, bioinformatics analysis was carried out on the data after the establishment of the database, and the analysis contents were mainly quantitative analysis of package identification, differential expression analysis and functional analysis.

### Animal experiment

This study was approved by the Laboratory Animal Welfare and Ethics Committee of the Ministry of Medicine (ethics number AHQU-MAL20220805). All methods were carried out in accordance with relevant regulations and ARRIVE guidelines. A total of 20 BALB/c nude mice, 5-week-old males, were purchased from Beijing Vital River Laboratory Animal Technology Co., Ltd. After the nude mice were stable in the SPF animal feeding environment for one week, the nude mice were subcutaneously injected with 5 × 10^6^ AGS cells into the subcutaneous region located in the right axilla. When the tumor volume was between 40 and 50 mm^3^, the nude mice were randomly assigned to one of five groups: a negative control group (DMSO and solvent), positive control group (5-Fu, 10 mg/kg or 25 mg/kg), or a treatment group (NTP, 10 mg/kg or 20 mg/kg), with 4 mice in each group. A total of 12 BALB/c nude mice were stable in the SPF animal feeding environment for one week, and 2.5 × 10^5^ MFC cells were injected into the subcutaneous region located on the right hind limb. When the tumor volume reached 50–80 mm^3^, the nude mice were randomly divided into three groups: the control group, positive control group (5-FU, 10 mg/kg) and drug treatment group (NTP, 20 mg/kg), with 4 mice in each group. The drug was injected intraperitoneally every two days. The long axis (L) and vertical axis (R) of the tumors were measured and recorded before each drug injection. Volume (V) calculation method: V = 0.5 × L × *R*^2^. The total duration of growth in the AGS tumor model was 33 days, which included 7 days for environmental adaptation, 10 days for growth after AGS cell injection and subsequent grouping, and 16 days for drug treatment (with 8 drug injections). The total duration of the MFC tumor model was 25 days, consisting of 7 days for environmental adaptation, 4 days for growth after MFC cell injection and subsequent grouping, and 14 days for drug treatment (with 7 drug injections).

### H&E staining

The vital organs were removed and fixed in 4% paraformaldehyde. The vital organs were embedded in paraffin, and frozen sections were stained with hematoxylin and eosin. The slides were dehydrated and sealed after staining. The images were collected after preparation.

### Statistical analysis

The data were statistically analyzed using GraphPad Prism 8. The data are expressed as the mean ± standard deviation (SD) of three or more replicates. Differences between two groups were analyzed by Student’s t test. Comparisons among multiple groups were analyzed by one-way ANOVA. P < 0.05 was considered to indicate statistical significance. * p < 0.05, ** p < 0.01, *** p < 0.001.

### Supplementary Information

Supplementary Information 1.

Supplementary Information 2.

## Data Availability

The datasets generated during and/or analyzed during the current study are available from the corresponding author upon reasonable request.

## Abbreviations

NTP: Nortriptyline hydrochloride
GPX4: Glutathione peroxidase 4
Keap1: Kelch-like ECH-associated protein 1
Nrf2: NF-E2-related factor 2
HO-1: Haem oxygenase 1
NQO1: NAD(P)H quinone dehydrogenase 1
ROS: Reactive oxygen species
GSH: Glutathione
MMP: Mitochondrial membrane potential
OS: Oxidative stress
MDA: Malondialdehyde
NAC: N-acetylcysteine
