# Supplementary material for: Nortriptyline hydrochloride, a potential candidate for drug repurposing, inhibits gastric cancer by inducing oxidative stress by triggering the Keap1-Nrf2 pathway
Source: Sci Rep. 2024 Mar 13;14:6050. doi: 10.1038/s41598-024-56431-5 (PMC10937941; doi:10.1038/s41598-024-56431-5)
Supplement: Supplementary file 1 — Supplementary Information 1. [file 41598_2024_56431_MOESM1_ESM.docx]

**Supplementary figures and table**


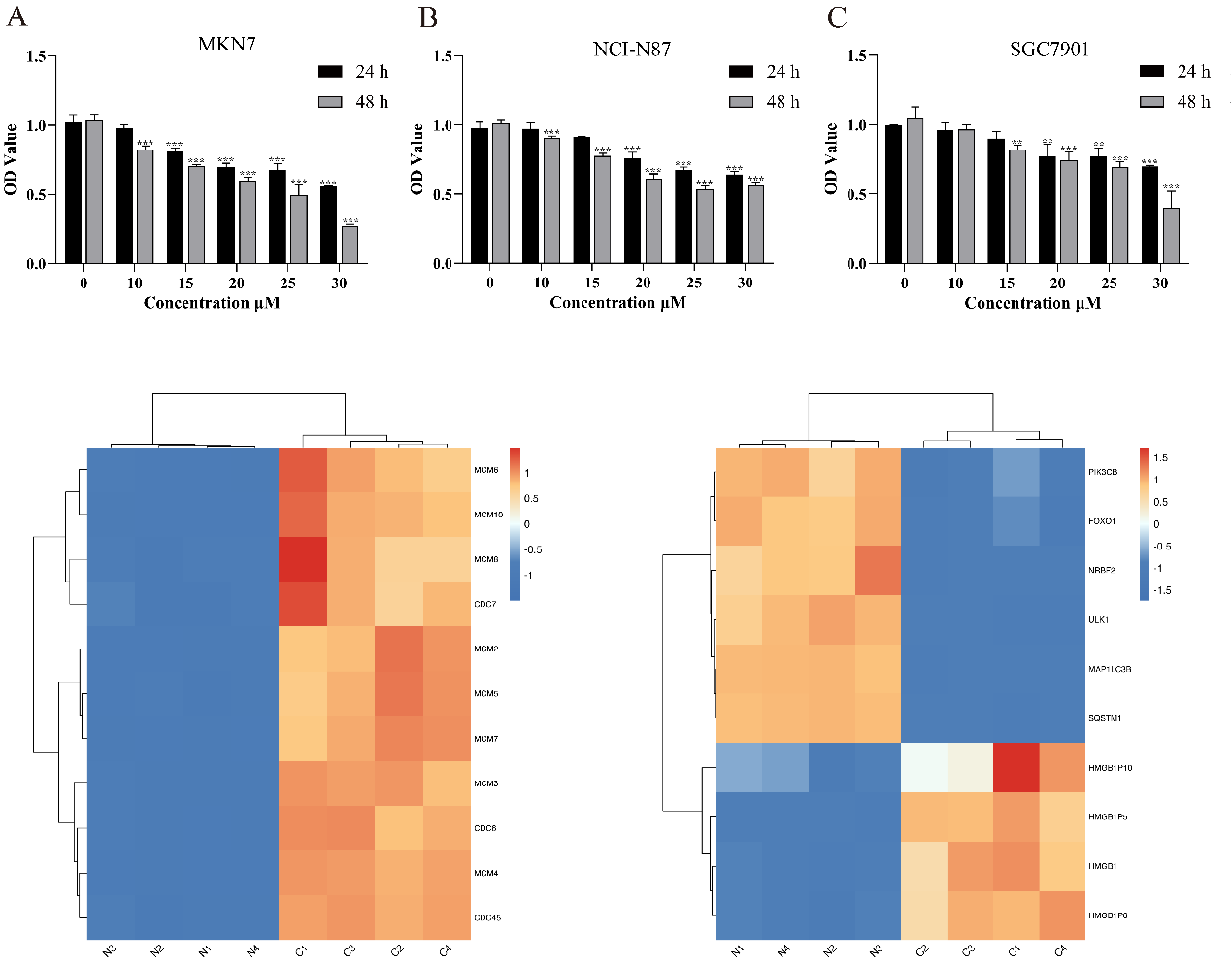


**Figure S1. (A-C)** MTT assay results of cell viability in MKN7, NCI-N87, and SGC7901 cells


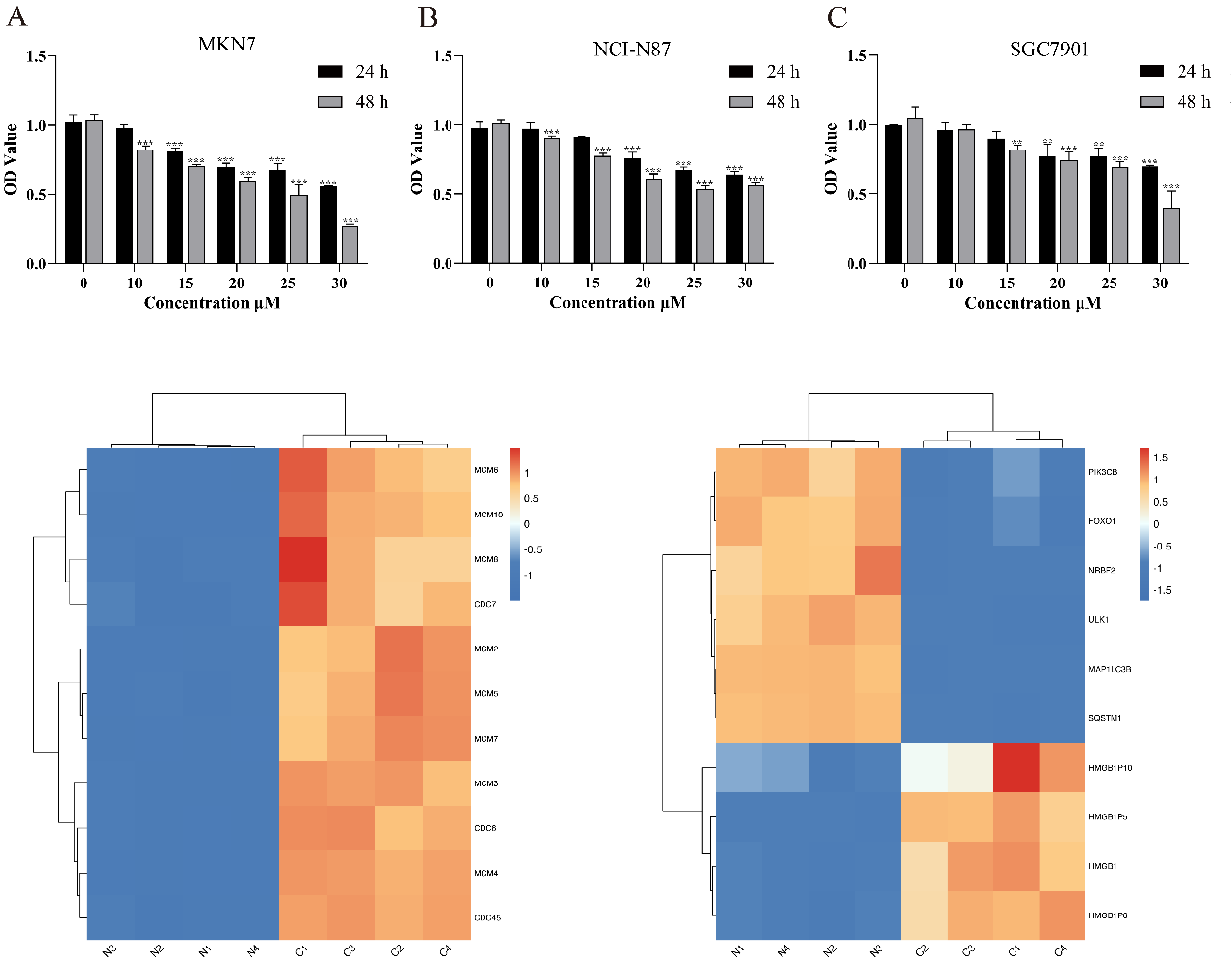


**Figure S2.** Cluster heatmap of genes related to cell proliferation in transcriptome data. C1-C4 represents the control group, and N1-N4 represents the NTP group.

**Supplementary Table 1**

**Blood Cell Analyzer Test Report**

Instrument Name: Mindray Veterinary Fully Automatic Blood Cell Analyzer Model: BC-5000vet

Mode: Whole Blood

NTP20-1

| Parameter | abbreviation | Result | Unit | Range |
| --- | --- | --- | --- | --- |
| White blood cell count | WBC | 12.90 | 10^9/L | 0.80 - 10.60 |
| Number of neutrophils | Neu# | 6.00 | 10^9/L | 0.23 - 3.60 |
| Lymphocyte count | Lym# | 2.23 | 10^9/L | 0.60 - 8.90 |
| Number of monocytes | Mon# | 4.36 | 10^9/L | 0.04 - 1.40 |
| Number of eosinophils | Eos# | 0.28 | 10^9/L | 0.00 - 0.51 |
| Number of basophils | Bas# | 0.03 | 10^9/L | 0.00 - 0.12 |
| Neutrophil percentage | Neu% | 46.5 | % | 6.5 - 50.0 |
| Lymphocyte percentage | Lym% | 17.3 | % | 40.0 - 92.0 |
| Percentage of monocytes | Mon% | 33.8 | % | 0.9 - 18.0 |
| Percentage of eosinophils | Eos% | 2.2 | % | 0.0 - 7.5 |
| Percentage of basophils | Bas% | 0.2 | % | 0.0 - 1.5 |
| Red blood cell count | RBC | 8.26 | 10^12/L | 6.50 - 11.50 |
| hemoglobin | HGB | 128 | g/L | 110 - 165 |
| Erythrocytosis | HCT | 43.4 | % | 35.0 - 55.0 |
| Average red blood cell volume | MCV | 52.6 | fL | 41.0 - 55.0 |
| Average hemoglobin content of red blood cells | MCH | 15.5 | pg | 13.0 - 18.0 |
| Mean corpuscular hemoglobin concentration | MCHC | 295 | g/L | 300 - 360 |
| Coefficient of variation of red blood cell distribution width | RDW-CV | 16.7 | % | 12.0 - 19.0 |
| Standard deviation of red blood cell distribution width | RDW-SD | 35.9 | fL | 23.0 - 39.0 |
| Platelet count | PLT | 650 | 10^9/L | 400 - 1600 |
| Average platelet volume | MPV | 5.9 | fL | 4.0 - 6.2 |
| Platelet distribution width | PDW | 16.0 |  | 12.0 - 17.5 |
| Platelet hematocrit | PCT | 0.383 | % | 0.100 - 0.780 |

NTP20-2

| Parameter | abbreviation | Result | Unit | Range |
| --- | --- | --- | --- | --- |
| White blood cell count | WBC | 14.19 | 10^9/L | 0.80 - 10.60 |
| Number of neutrophils | Neu# | 5.05 | 10^9/L | 0.23 - 3.60 |
| Lymphocyte count | Lym# | 1.62 | 10^9/L | 0.60 - 8.90 |
| Number of monocytes | Mon# | 7.00 | 10^9/L | 0.04 - 1.40 |
| Number of eosinophils | Eos# | 0.48 | 10^9/L | 0.00 - 0.51 |
| Number of basophils | Bas# | 0.04 | 10^9/L | 0.00 - 0.12 |
| Neutrophil percentage | Neu% | 35.6 | % | 6.5 - 50.0 |
| Lymphocyte percentage | Lym% | 11.4 | % | 40.0 - 92.0 |
| Percentage of monocytes | Mon% | 49.3 | % | 0.9 - 18.0 |
| Percentage of eosinophils | Eos% | 3.4 | % | 0.0 - 7.5 |
| Percentage of basophils | Bas% | 0.3 | % | 0.0 - 1.5 |
| Red blood cell count | RBC | 8.46 | 10^12/L | 6.50 - 11.50 |
| hemoglobin | HGB | 131 | g/L | 110 - 165 |
| Erythrocytosis | HCT | 45.3 | % | 35.0 - 55.0 |
| Average red blood cell volume | MCV | 53.5 | fL | 41.0 - 55.0 |
| Average hemoglobin content of red blood cells | MCH | 15.5 | pg | 13.0 - 18.0 |
| Mean corpuscular hemoglobin concentration | MCHC | 290 | g/L | 300 - 360 |
| Coefficient of variation of red blood cell distribution width | RDW-CV | 16.9 | % | 12.0 - 19.0 |
| Standard deviation of red blood cell distribution width | RDW-SD | 37.0 | fL | 23.0 - 39.0 |
| Platelet count | PLT | 508 | 10^9/L | 400 - 1600 |
| Average platelet volume | MPV | 6.0 | fL | 4.0 - 6.2 |
| Platelet distribution width | PDW | 15.7 |  | 12.0 - 17.5 |
| Platelet hematocrit | PCT | 0.307 | % | 0.100 - 0.780 |

NTP20-3

| Parameter | abbreviation | Result | Unit | Range |
| --- | --- | --- | --- | --- |
| White blood cell count | WBC | 12.28 | 10^9/L | 0.80 - 10.60 |
| Number of neutrophils | Neu# | 5.95 | 10^9/L | 0.23 - 3.60 |
| Lymphocyte count | Lym# | 1.82 | 10^9/L | 0.60 - 8.90 |
| Number of monocytes | Mon# | 4.23 | 10^9/L | 0.04 - 1.40 |
| Number of eosinophils | Eos# | 0.23 | 10^9/L | 0.00 - 0.51 |
| Number of basophils | Bas# | 0.05 | 10^9/L | 0.00 - 0.12 |
| Neutrophil percentage | Neu% | 48.4 | % | 6.5 - 50.0 |
| Lymphocyte percentage | Lym% | 14.9 | % | 40.0 - 92.0 |
| Percentage of monocytes | Mon% | 34.4 | % | 0.9 - 18.0 |
| Percentage of eosinophils | Eos% | 1.9 | % | 0.0 - 7.5 |
| Percentage of basophils | Bas% | 0.4 | % | 0.0 - 1.5 |
| Red blood cell count | RBC | 9.30 | 10^12/L | 6.50 - 11.50 |
| hemoglobin | HGB | 147 | g/L | 110 - 165 |
| Erythrocytosis | HCT | 48.0 | % | 35.0 - 55.0 |
| Average red blood cell volume | MCV | 51.7 | fL | 41.0 - 55.0 |
| Average hemoglobin content of red blood cells | MCH | 15.8 | pg | 13.0 - 18.0 |
| Mean corpuscular hemoglobin concentration | MCHC | 306 | g/L | 300 - 360 |
| Coefficient of variation of red blood cell distribution width | RDW-CV | 16.2 | % | 12.0 - 19.0 |
| Standard deviation of red blood cell distribution width | RDW-SD | 34.3 | fL | 23.0 - 39.0 |
| Platelet count | PLT | 673 | 10^9/L | 400 - 1600 |
| Average platelet volume | MPV | 7.2 | fL | 4.0 - 6.2 |
| Platelet distribution width | PDW | 15.7 |  | 12.0 - 17.5 |
| Platelet hematocrit | PCT | 0.486 | % | 0.100 - 0.780 |

NTP20-4

| Parameter | abbreviation | Result | Unit | Range |
| --- | --- | --- | --- | --- |
| White blood cell count | WBC | 14.53 | 10^9/L | 0.80 - 10.60 |
| Number of neutrophils | Neu# | 5.11 | 10^9/L | 0.23 - 3.60 |
| Lymphocyte count | Lym# | 3.11 | 10^9/L | 0.60 - 8.90 |
| Number of monocytes | Mon# | 6.06 | 10^9/L | 0.04 - 1.40 |
| Number of eosinophils | Eos# | 0.24 | 10^9/L | 0.00 - 0.51 |
| Number of basophils | Bas# | 0.01 | 10^9/L | 0.00 - 0.12 |
| Neutrophil percentage | Neu% | 35.2 | % | 6.5 - 50.0 |
| Lymphocyte percentage | Lym% | 21.4 | % | 40.0 - 92.0 |
| Percentage of monocytes | Mon% | 41.7 | % | 0.9 - 18.0 |
| Percentage of eosinophils | Eos% | 1.6 | % | 0.0 - 7.5 |
| Percentage of basophils | Bas% | 0.1 | % | 0.0 - 1.5 |
| Red blood cell count | RBC | 8.75 | 10^12/L | 6.50 - 11.50 |
| hemoglobin | HGB | 139 | g/L | 110 - 165 |
| Erythrocytosis | HCT | 44.6 | % | 35.0 - 55.0 |
| Average red blood cell volume | MCV | 51.0 | fL | 41.0 - 55.0 |
| Average hemoglobin content of red blood cells | MCH | 15.9 | pg | 13.0 - 18.0 |
| Mean corpuscular hemoglobin concentration | MCHC | 311 | g/L | 300 - 360 |
| Coefficient of variation of red blood cell distribution width | RDW-CV | 16.5 | % | 12.0 - 19.0 |
| Standard deviation of red blood cell distribution width | RDW-SD | 34.7 | fL | 23.0 - 39.0 |
| Platelet count | PLT | 959 | 10^9/L | 400 - 1600 |
| Average platelet volume | MPV | 6.1 | fL | 4.0 - 6.2 |
| Platelet distribution width | PDW | 16.3 |  | 12.0 - 17.5 |
| Platelet hematocrit | PCT | 0.580 | % | 0.100 - 0.780 |

Control-1

| Parameter | abbreviation | Result | Unit | Range |
| --- | --- | --- | --- | --- |
| White blood cell count | WBC | 10.43 | 10^9/L | 0.80 - 10.60 |
| Number of neutrophils | Neu# | 3.59 | 10^9/L | 0.23 - 3.60 |
| Lymphocyte count | Lym# | 1.70 | 10^9/L | 0.60 - 8.90 |
| Number of monocytes | Mon# | 4.74 | 10^9/L | 0.04 - 1.40 |
| Number of eosinophils | Eos# | 0.37 | 10^9/L | 0.00 - 0.51 |
| Number of basophils | Bas# | 0.03 | 10^9/L | 0.00 - 0.12 |
| Neutrophil percentage | Neu% | 34.4 | % | 6.5 - 50.0 |
| Lymphocyte percentage | Lym% | 16.3 | % | 40.0 - 92.0 |
| Percentage of monocytes | Mon% | 45.5 | % | 0.9 - 18.0 |
| Percentage of eosinophils | Eos% | 3.5 | % | 0.0 - 7.5 |
| Percentage of basophils | Bas% | 0.3 | % | 0.0 - 1.5 |
| Red blood cell count | RBC | 8.85 | 10^12/L | 6.50 - 11.50 |
| hemoglobin | HGB | 141 | g/L | 110 - 165 |
| Erythrocytosis | HCT | 48.2 | % | 35.0 - 55.0 |
| Average red blood cell volume | MCV | 54.5 | fL | 41.0 - 55.0 |
| Average hemoglobin content of red blood cells | MCH | 15.9 | pg | 13.0 - 18.0 |
| Mean corpuscular hemoglobin concentration | MCHC | 292 | g/L | 300 - 360 |
| Coefficient of variation of red blood cell distribution width | RDW-CV | 17.0 | % | 12.0 - 19.0 |
| Standard deviation of red blood cell distribution width | RDW-SD | 38.0 | fL | 23.0 - 39.0 |
| Platelet count | PLT | 472 | 10^9/L | 400 - 1600 |
| Average platelet volume | MPV | 6.3 | fL | 4.0 - 6.2 |
| Platelet distribution width | PDW | 15.9 |  | 12.0 - 17.5 |
| Platelet hematocrit | PCT | 0.296 | % | 0.100 - 0.780 |

Control-2

| Parameter | abbreviation | Result | Unit | Range |
| --- | --- | --- | --- | --- |
| White blood cell count | WBC | 12.35 | 10^9/L | 0.80 - 10.60 |
| Number of neutrophils | Neu# | 3.61 | 10^9/L | 0.23 - 3.60 |
| Lymphocyte count | Lym# | 4.42 | 10^9/L | 0.60 - 8.90 |
| Number of monocytes | Mon# | 3.98 | 10^9/L | 0.04 - 1.40 |
| Number of eosinophils | Eos# | 0.30 | 10^9/L | 0.00 - 0.51 |
| Number of basophils | Bas# | 0.04 | 10^9/L | 0.00 - 0.12 |
| Neutrophil percentage | Neu% | 29.3 | % | 6.5 - 50.0 |
| Lymphocyte percentage | Lym% | 35.8 | % | 40.0 - 92.0 |
| Percentage of monocytes | Mon% | 32.2 | % | 0.9 - 18.0 |
| Percentage of eosinophils | Eos% | 2.4 | % | 0.0 - 7.5 |
| Percentage of basophils | Bas% | 0.3 | % | 0.0 - 1.5 |
| Red blood cell count | RBC | 9.16 | 10^12/L | 6.50 - 11.50 |
| hemoglobin | HGB | 143 | g/L | 110 - 165 |
| Erythrocytosis | HCT | 47.5 | % | 35.0 - 55.0 |
| Average red blood cell volume | MCV | 51.8 | fL | 41.0 - 55.0 |
| Average hemoglobin content of red blood cells | MCH | 15.6 | pg | 13.0 - 18.0 |
| Mean corpuscular hemoglobin concentration | MCHC | 300 | g/L | 300 - 360 |
| Coefficient of variation of red blood cell distribution width | RDW-CV | 15.8 | % | 12.0 - 19.0 |
| Standard deviation of red blood cell distribution width | RDW-SD | 33.7 | fL | 23.0 - 39.0 |
| Platelet count | PLT | 838 | 10^9/L | 400 - 1600 |
| Average platelet volume | MPV | 6.5 | fL | 4.0 - 6.2 |
| Platelet distribution width | PDW | 16.0 |  | 12.0 - 17.5 |
| Platelet hematocrit | PCT | 0.542 | % | 0.100 - 0.780 |

Control-3

| Parameter | abbreviation | Result | Unit | Range |
| --- | --- | --- | --- | --- |
| White blood cell count | WBC | 6.49 | 10^9/L | 0.80 - 10.60 |
| Number of neutrophils | Neu# | 2.50 | 10^9/L | 0.23 - 3.60 |
| Lymphocyte count | Lym# | 1.30 | 10^9/L | 0.60 - 8.90 |
| Number of monocytes | Mon# | 2.36 | 10^9/L | 0.04 - 1.40 |
| Number of eosinophils | Eos# | 0.32 | 10^9/L | 0.00 - 0.51 |
| Number of basophils | Bas# | 0.01 | 10^9/L | 0.00 - 0.12 |
| Neutrophil percentage | Neu% | 38.5 | % | 6.5 - 50.0 |
| Lymphocyte percentage | Lym% | 20.1 | % | 40.0 - 92.0 |
| Percentage of monocytes | Mon% | 36.3 | % | 0.9 - 18.0 |
| Percentage of eosinophils | Eos% | 4.9 | % | 0.0 - 7.5 |
| Percentage of basophils | Bas% | 0.2 | % | 0.0 - 1.5 |
| Red blood cell count | RBC | 5.33 | 10^12/L | 6.50 - 11.50 |
| hemoglobin | HGB | 83 | g/L | 110 - 165 |
| Erythrocytosis | HCT | 27.7 | % | 35.0 - 55.0 |
| Average red blood cell volume | MCV | 52.0 | fL | 41.0 - 55.0 |
| Average hemoglobin content of red blood cells | MCH | 15.6 | pg | 13.0 - 18.0 |
| Mean corpuscular hemoglobin concentration | MCHC | 299 | g/L | 300 - 360 |
| Coefficient of variation of red blood cell distribution width | RDW-CV | 16.5 | % | 12.0 - 19.0 |
| Standard deviation of red blood cell distribution width | RDW-SD | 35.0 | fL | 23.0 - 39.0 |
| Platelet count | PLT | 480 | 10^9/L | 400 - 1600 |
| Average platelet volume | MPV | 6.5 | fL | 4.0 - 6.2 |
| Platelet distribution width | PDW | 16.1 |  | 12.0 - 17.5 |
| Platelet hematocrit | PCT | 0.314 | % | 0.100 - 0.780 |

Control-4

| Parameter | abbreviation | Result | Unit | Range |
| --- | --- | --- | --- | --- |
| White blood cell count | WBC | 13.23 | 10^9/L | 0.80 - 10.60 |
| Number of neutrophils | Neu# | 4.05 | 10^9/L | 0.23 - 3.60 |
| Lymphocyte count | Lym# | 1.95 | 10^9/L | 0.60 - 8.90 |
| Number of monocytes | Mon# | 6.52 | 10^9/L | 0.04 - 1.40 |
| Number of eosinophils | Eos# | 0.69 | 10^9/L | 0.00 - 0.51 |
| Number of basophils | Bas# | 0.02 | 10^9/L | 0.00 - 0.12 |
| Neutrophil percentage | Neu% | 30.6 | % | 6.5 - 50.0 |
| Lymphocyte percentage | Lym% | 14.7 | % | 40.0 - 92.0 |
| Percentage of monocytes | Mon% | 49.3 | % | 0.9 - 18.0 |
| Percentage of eosinophils | Eos% | 5.2 | % | 0.0 - 7.5 |
| Percentage of basophils | Bas% | 0.2 | % | 0.0 - 1.5 |
| Red blood cell count | RBC | 8.91 | 10^12/L | 6.50 - 11.50 |
| hemoglobin | HGB | 141 | g/L | 110 - 165 |
| Erythrocytosis | HCT | 45.3 | % | 35.0 - 55.0 |
| Average red blood cell volume | MCV | 50.8 | fL | 41.0 - 55.0 |
| Average hemoglobin content of red blood cells | MCH | 15.8 | pg | 13.0 - 18.0 |
| Mean corpuscular hemoglobin concentration | MCHC | 311 | g/L | 300 - 360 |
| Coefficient of variation of red blood cell distribution width | RDW-CV | 16.7 | % | 12.0 - 19.0 |
| Standard deviation of red blood cell distribution width | RDW-SD | 35.0 | fL | 23.0 - 39.0 |
| Platelet count | PLT | 981 | 10^9/L | 400 - 1600 |
| Average platelet volume | MPV | 6.5 | fL | 4.0 - 6.2 |
| Platelet distribution width | PDW | 16.3 |  | 12.0 - 17.5 |
| Platelet hematocrit | PCT | 0.637 | % | 0.100 - 0.780 |

5-Fu-1

| Parameter | abbreviation | Result | Unit | Range |
| --- | --- | --- | --- | --- |
| White blood cell count | WBC | 8.24 | 10^9/L | 0.80 - 10.60 |
| Number of neutrophils | Neu# | 3.01 | 10^9/L | 0.23 - 3.60 |
| Lymphocyte count | Lym# | 1.35 | 10^9/L | 0.60 - 8.90 |
| Number of monocytes | Mon# | 3.57 | 10^9/L | 0.04 - 1.40 |
| Number of eosinophils | Eos# | 0.28 | 10^9/L | 0.00 - 0.51 |
| Number of basophils | Bas# | 0.03 | 10^9/L | 0.00 - 0.12 |
| Neutrophil percentage | Neu% | 36.5 | % | 6.5 - 50.0 |
| Lymphocyte percentage | Lym% | 16.2 | % | 40.0 - 92.0 |
| Percentage of monocytes | Mon% | 43.4 | % | 0.9 - 18.0 |
| Percentage of eosinophils | Eos% | 3.5 | % | 0.0 - 7.5 |
| Percentage of basophils | Bas% | 0.4 | % | 0.0 - 1.5 |
| Red blood cell count | RBC | 7.96 | 10^12/L | 6.50 - 11.50 |
| hemoglobin | HGB | 125 | g/L | 110 - 165 |
| Erythrocytosis | HCT | 43.1 | % | 35.0 - 55.0 |
| Average red blood cell volume | MCV | 54.1 | fL | 41.0 - 55.0 |
| Average hemoglobin content of red blood cells | MCH | 15.7 | pg | 13.0 - 18.0 |
| Mean corpuscular hemoglobin concentration | MCHC | 291 | g/L | 300 - 360 |
| Coefficient of variation of red blood cell distribution width | RDW-CV | 18.1 | % | 12.0 - 19.0 |
| Standard deviation of red blood cell distribution width | RDW-SD | 39.9 | fL | 23.0 - 39.0 |
| Platelet count | PLT | 417 | 10^9/L | 400 - 1600 |
| Average platelet volume | MPV | 5.9 | fL | 4.0 - 6.2 |
| Platelet distribution width | PDW | 15.9 |  | 12.0 - 17.5 |
| Platelet hematocrit | PCT | 0.245 | % | 0.100 - 0.780 |

5-Fu-2

| Parameter | abbreviation | Result | Unit | Range |
| --- | --- | --- | --- | --- |
| White blood cell count | WBC | 9.68 | 10^9/L | 0.80 - 10.60 |
| Number of neutrophils | Neu# | 2.55 | 10^9/L | 0.23 - 3.60 |
| Lymphocyte count | Lym# | 2.08 | 10^9/L | 0.60 - 8.90 |
| Number of monocytes | Mon# | 4.38 | 10^9/L | 0.04 - 1.40 |
| Number of eosinophils | Eos# | 0.64 | 10^9/L | 0.00 - 0.51 |
| Number of basophils | Bas# | 0.03 | 10^9/L | 0.00 - 0.12 |
| Neutrophil percentage | Neu% | 26.3 | % | 6.5 - 50.0 |
| Lymphocyte percentage | Lym% | 21.5 | % | 40.0 - 92.0 |
| Percentage of monocytes | Mon% | 45.3 | % | 0.9 - 18.0 |
| Percentage of eosinophils | Eos% | 6.6 | % | 0.0 - 7.5 |
| Percentage of basophils | Bas% | 0.3 | % | 0.0 - 1.5 |
| Red blood cell count | RBC | 7.06 | 10^12/L | 6.50 - 11.50 |
| hemoglobin | HGB | 115 | g/L | 110 - 165 |
| Erythrocytosis | HCT | 38.3 | % | 35.0 - 55.0 |
| Average red blood cell volume | MCV | 54.3 | fL | 41.0 - 55.0 |
| Average hemoglobin content of red blood cells | MCH | 16.2 | pg | 13.0 - 18.0 |
| Mean corpuscular hemoglobin concentration | MCHC | 299 | g/L | 300 - 360 |
| Coefficient of variation of red blood cell distribution width | RDW-CV | 17.6 | % | 12.0 - 19.0 |
| Standard deviation of red blood cell distribution width | RDW-SD | 39.0 | fL | 23.0 - 39.0 |
| Platelet count | PLT | 398 | 10^9/L | 400 - 1600 |
| Average platelet volume | MPV | 6.4 | fL | 4.0 - 6.2 |
| Platelet distribution width | PDW | 16.1 |  | 12.0 - 17.5 |
| Platelet hematocrit | PCT | 0.256 | % | 0.100 - 0.780 |

5-Fu-3

| Parameter | abbreviation | Result | Unit | Range |
| --- | --- | --- | --- | --- |
| White blood cell count | WBC | 10.49 | 10^9/L | 0.80 - 10.60 |
| Number of neutrophils | Neu# | 3.68 | 10^9/L | 0.23 - 3.60 |
| Lymphocyte count | Lym# | 1.82 | 10^9/L | 0.60 - 8.90 |
| Number of monocytes | Mon# | 4.63 | 10^9/L | 0.04 - 1.40 |
| Number of eosinophils | Eos# | 0.33 | 10^9/L | 0.00 - 0.51 |
| Number of basophils | Bas# | 0.03 | 10^9/L | 0.00 - 0.12 |
| Neutrophil percentage | Neu% | 35.1 | % | 6.5 - 50.0 |
| Lymphocyte percentage | Lym% | 17.4 | % | 40.0 - 92.0 |
| Percentage of monocytes | Mon% | 44.1 | % | 0.9 - 18.0 |
| Percentage of eosinophils | Eos% | 3.1 | % | 0.0 - 7.5 |
| Percentage of basophils | Bas% | 0.3 | % | 0.0 - 1.5 |
| Red blood cell count | RBC | 9.63 | 10^12/L | 6.50 - 11.50 |
| hemoglobin | HGB | 152 | g/L | 110 - 165 |
| Erythrocytosis | HCT | 49.3 | % | 35.0 - 55.0 |
| Average red blood cell volume | MCV | 51.2 | fL | 41.0 - 55.0 |
| Average hemoglobin content of red blood cells | MCH | 15.8 | pg | 13.0 - 18.0 |
| Mean corpuscular hemoglobin concentration | MCHC | 309 | g/L | 300 - 360 |
| Coefficient of variation of red blood cell distribution width | RDW-CV | 17.0 | % | 12.0 - 19.0 |
| Standard deviation of red blood cell distribution width | RDW-SD | 35.9 | fL | 23.0 - 39.0 |
| Platelet count | PLT | 981 | 10^9/L | 400 - 1600 |
| Average platelet volume | MPV | 6.1 | fL | 4.0 - 6.2 |
| Platelet distribution width | PDW | 16.2 |  | 12.0 - 17.5 |
| Platelet hematocrit | PCT | 0.599 | % | 0.100 - 0.780 |

5-Fu-4

| Parameter | abbreviation | Result | Unit | Range |
| --- | --- | --- | --- | --- |
| White blood cell count | WBC | 11.80 | 10^9/L | 0.80 - 10.60 |
| Number of neutrophils | Neu# | 3.30 | 10^9/L | 0.23 - 3.60 |
| Lymphocyte count | Lym# | 2.44 | 10^9/L | 0.60 - 8.90 |
| Number of monocytes | Mon# | 4.91 | 10^9/L | 0.04 - 1.40 |
| Number of eosinophils | Eos# | 1.12 | 10^9/L | 0.00 - 0.51 |
| Number of basophils | Bas# | 0.03 | 10^9/L | 0.00 - 0.12 |
| Neutrophil percentage | Neu% | 28.0 | % | 6.5 - 50.0 |
| Lymphocyte percentage | Lym% | 20.6 | % | 40.0 - 92.0 |
| Percentage of monocytes | Mon% | 41.6 | % | 0.9 - 18.0 |
| Percentage of eosinophils | Eos% | 9.5 | % | 0.0 - 7.5 |
| Percentage of basophils | Bas% | 0.3 | % | 0.0 - 1.5 |
| Red blood cell count | RBC | 8.81 | 10^12/L | 6.50 - 11.50 |
| hemoglobin | HGB | 138 | g/L | 110 - 165 |
| Erythrocytosis | HCT | 46.2 | % | 35.0 - 55.0 |
| Average red blood cell volume | MCV | 52.5 | fL | 41.0 - 55.0 |
| Average hemoglobin content of red blood cells | MCH | 15.7 | pg | 13.0 - 18.0 |
| Mean corpuscular hemoglobin concentration | MCHC | 299 | g/L | 300 - 360 |
| Coefficient of variation of red blood cell distribution width | RDW-CV | 16.5 | % | 12.0 - 19.0 |
| Standard deviation of red blood cell distribution width | RDW-SD | 35.7 | fL | 23.0 - 39.0 |
| Platelet count | PLT | 992 | 10^9/L | 400 - 1600 |
| Average platelet volume | MPV | 5.8 | fL | 4.0 - 6.2 |
| Platelet distribution width | PDW | 16.4 |  | 12.0 - 17.5 |
| Platelet hematocrit | PCT | 0.576 | % | 0.100 - 0.780 |
