# Supplementary figures and images for: Nortriptyline hydrochloride, a potential candidate for drug repurposing, inhibits gastric cancer by inducing oxidative stress by triggering the Keap1-Nrf2 pathway
Source: Sci Rep. 2024 Mar 13;14:6050. doi: 10.1038/s41598-024-56431-5 (PMC10937941; doi:10.1038/s41598-024-56431-5)

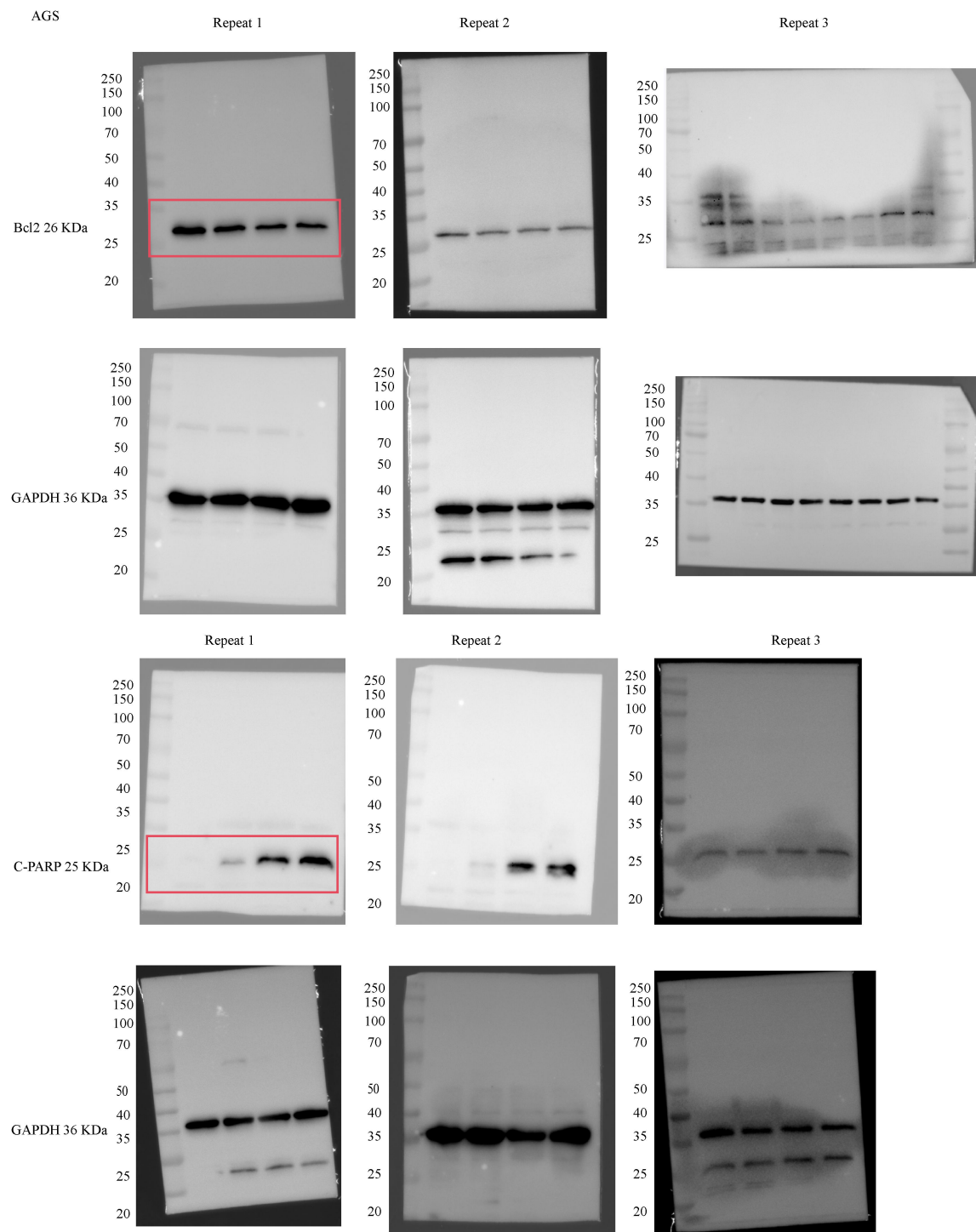

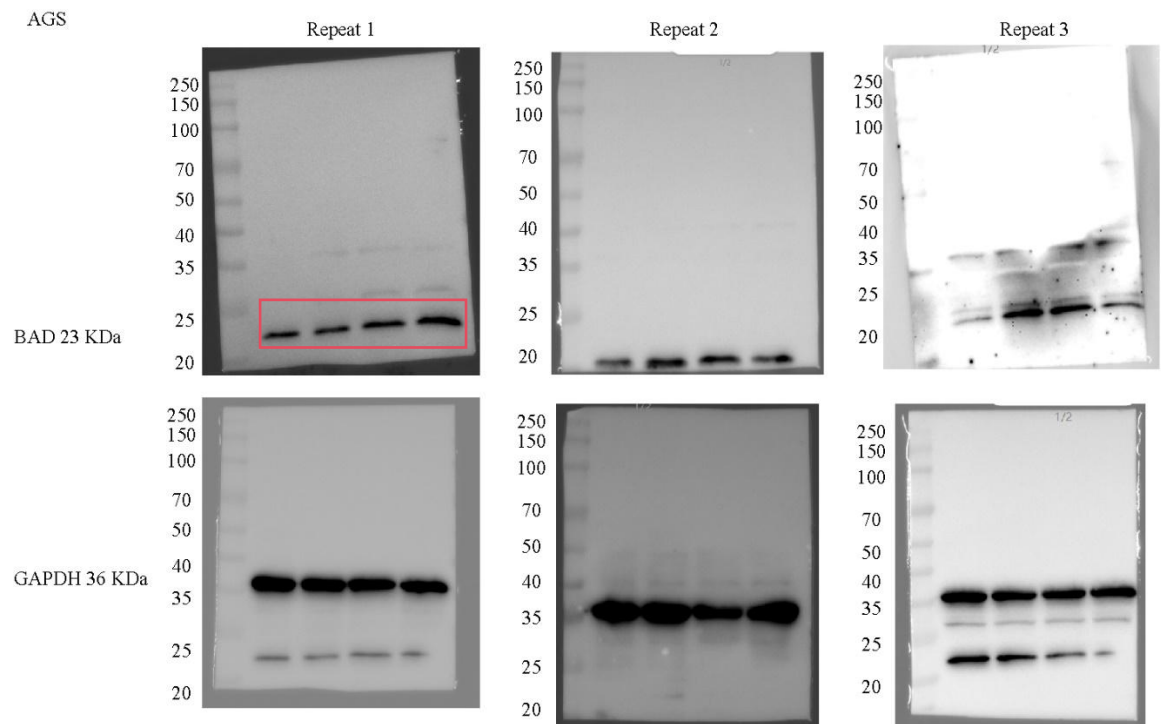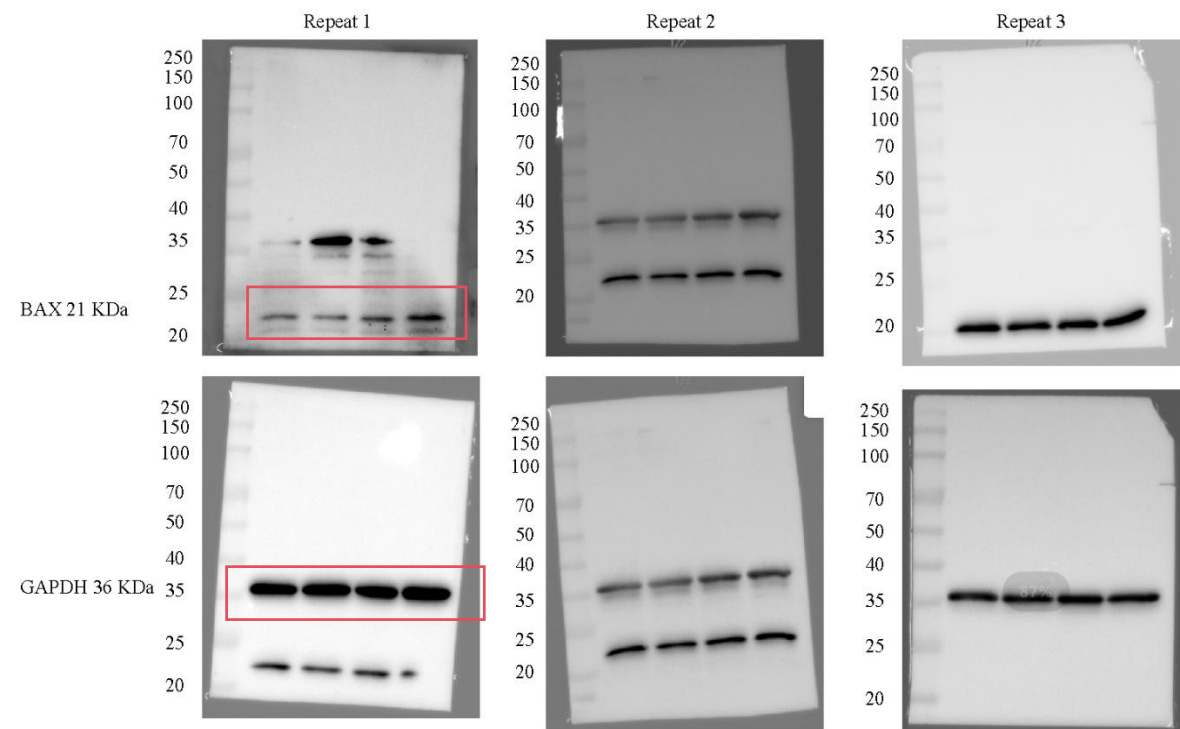

AGS

Repeat 1

Repeat 2

Repeat 3

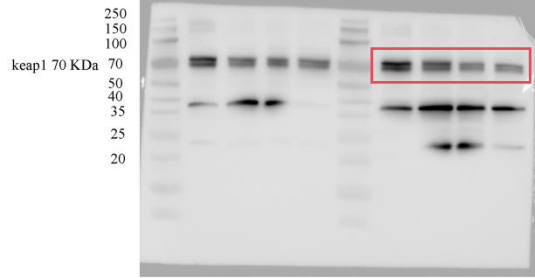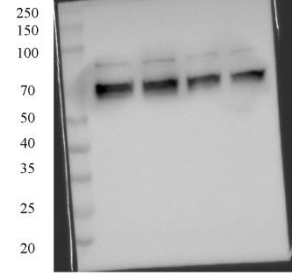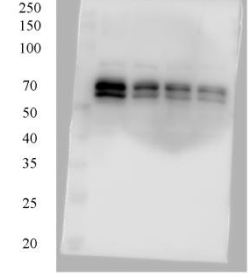

GAPDH 36 KDa

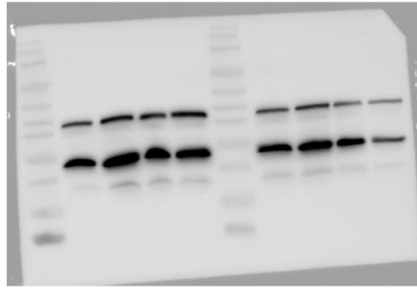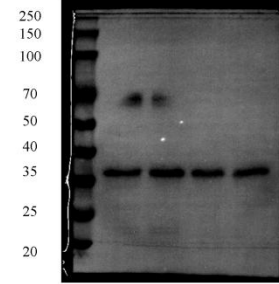

Repeat 1

Repeat 2

Repeat 3

Repeat 4

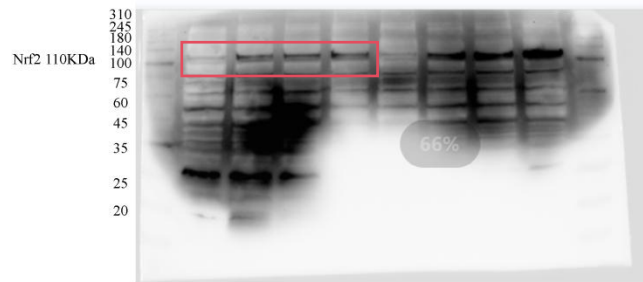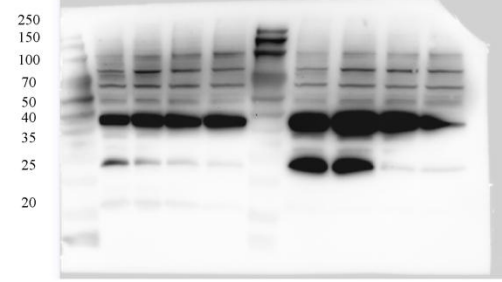

GAPDH 36 KDa

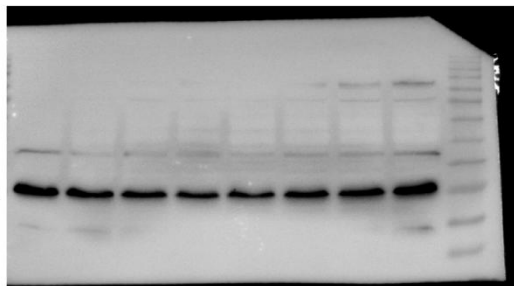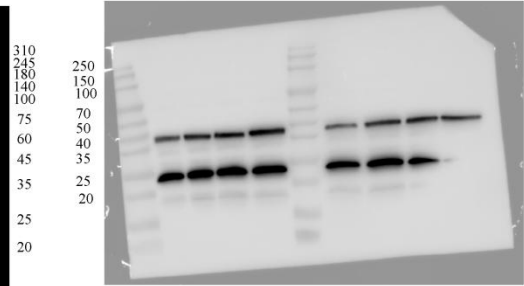

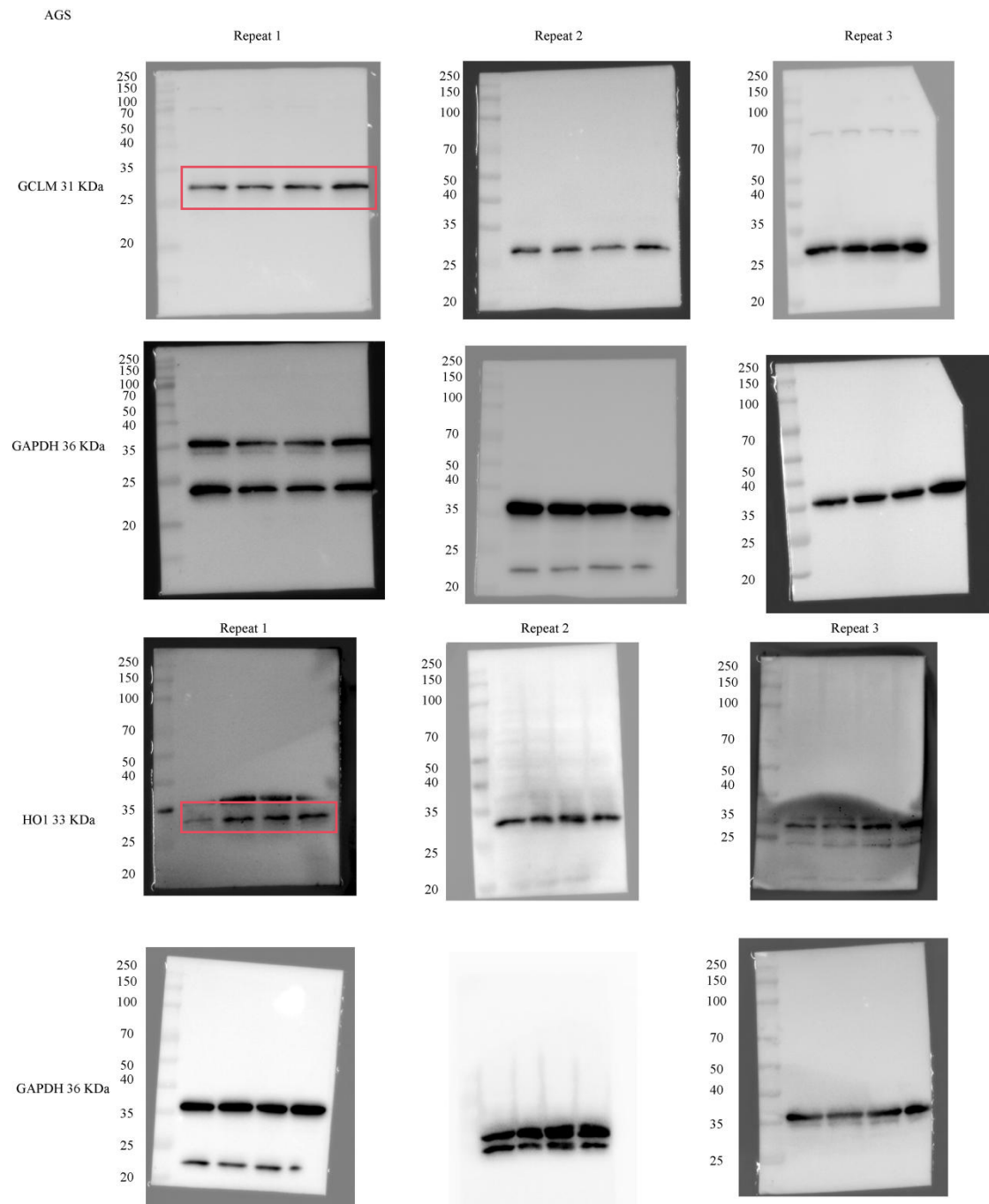

AGS

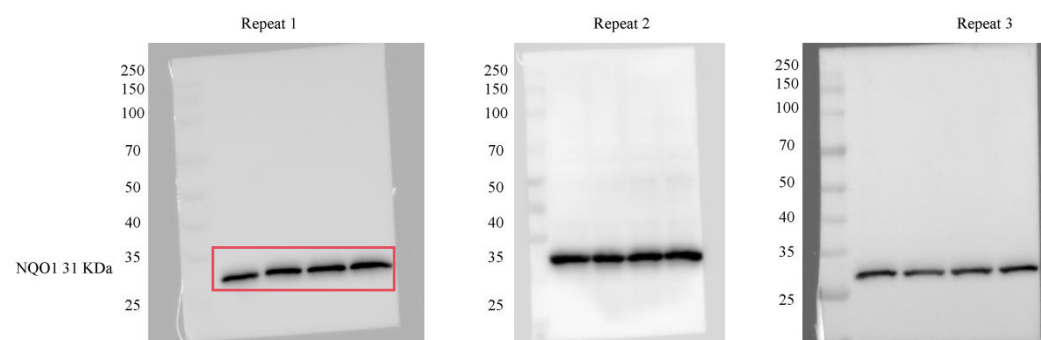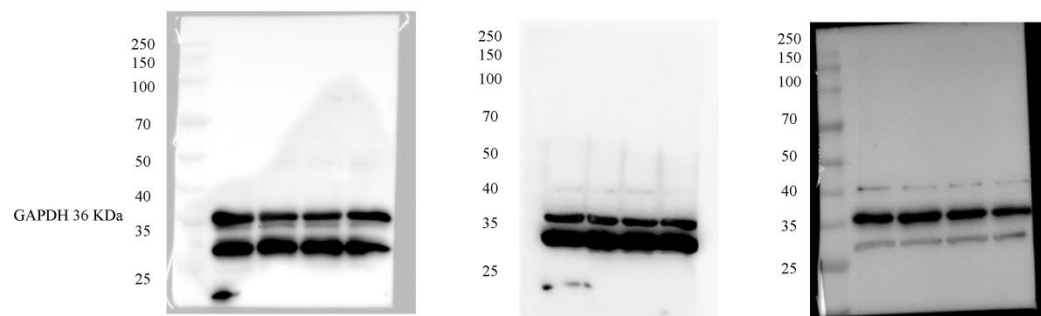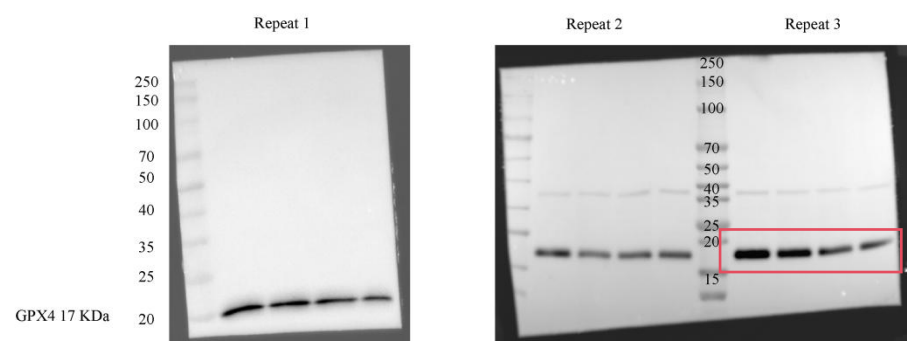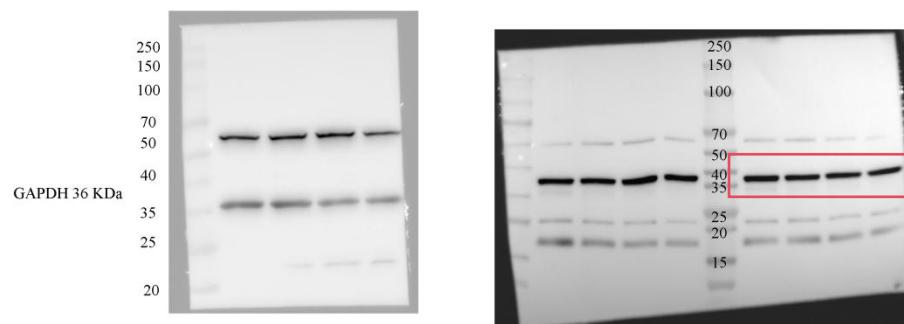

HGC27

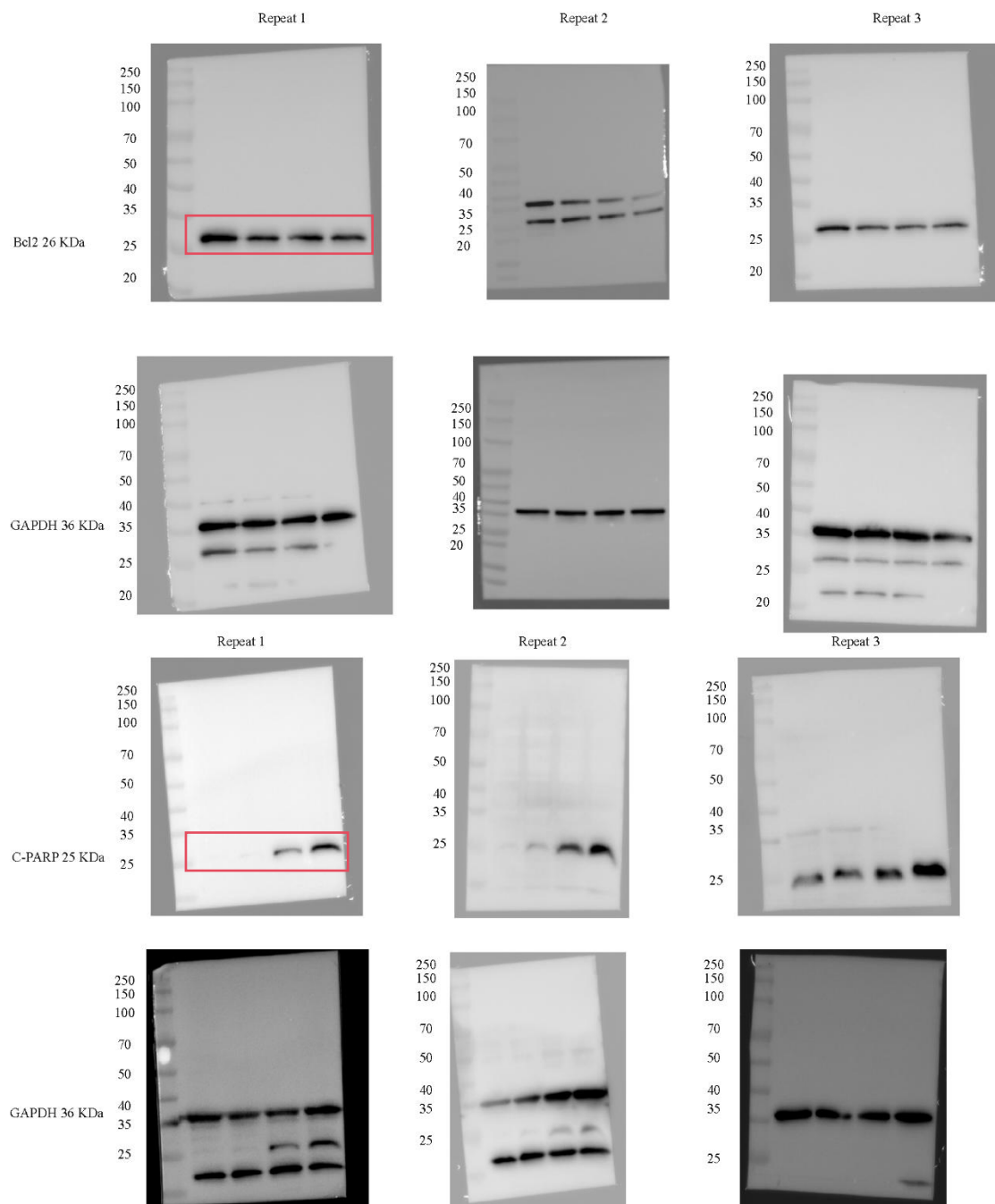

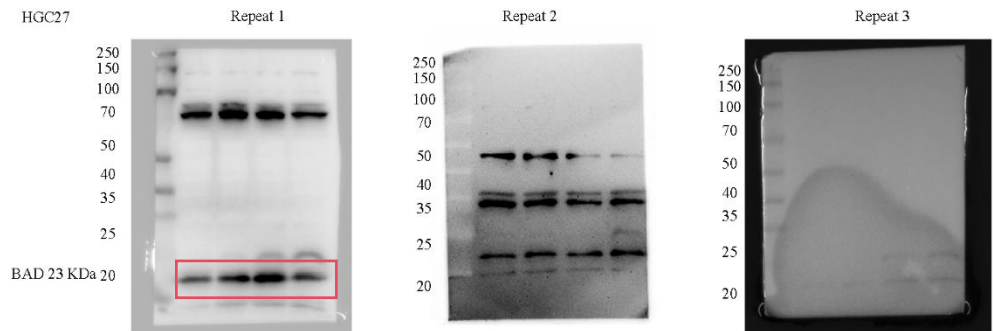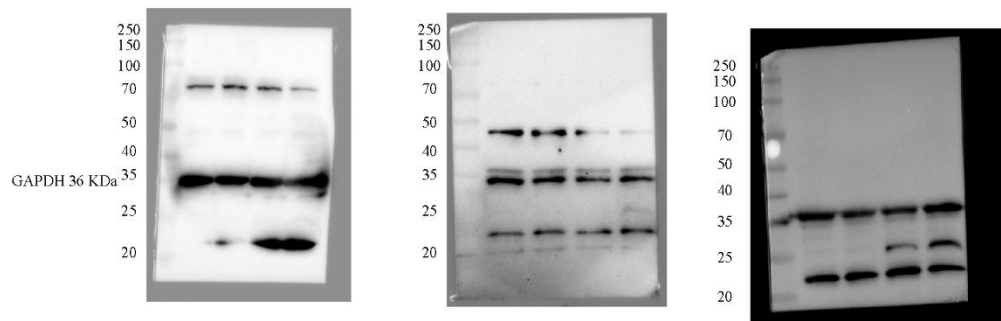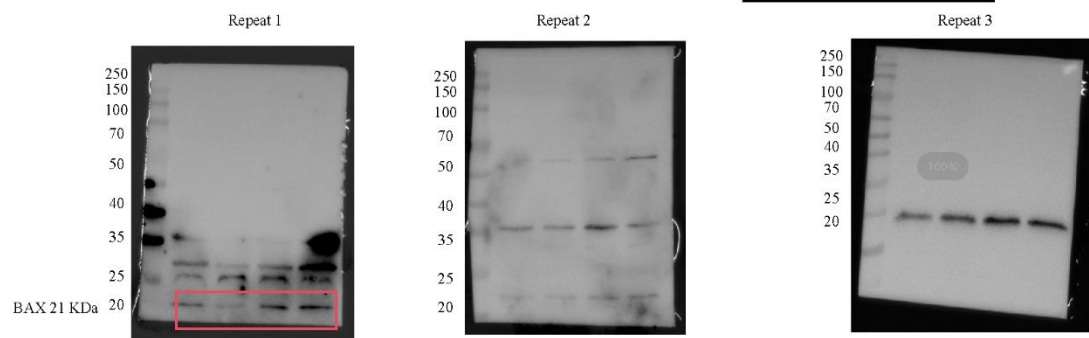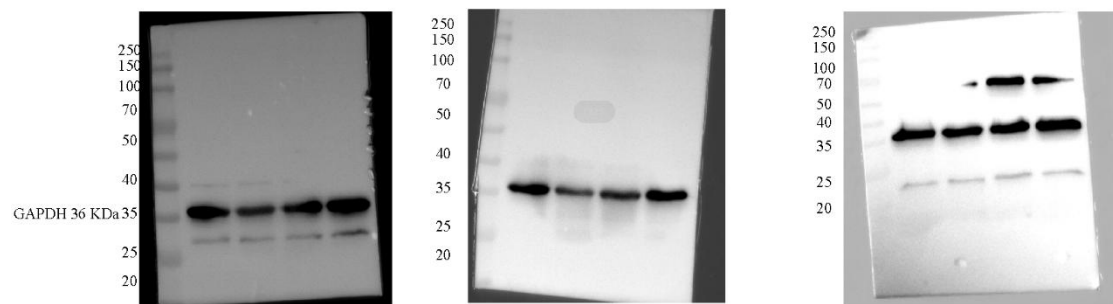

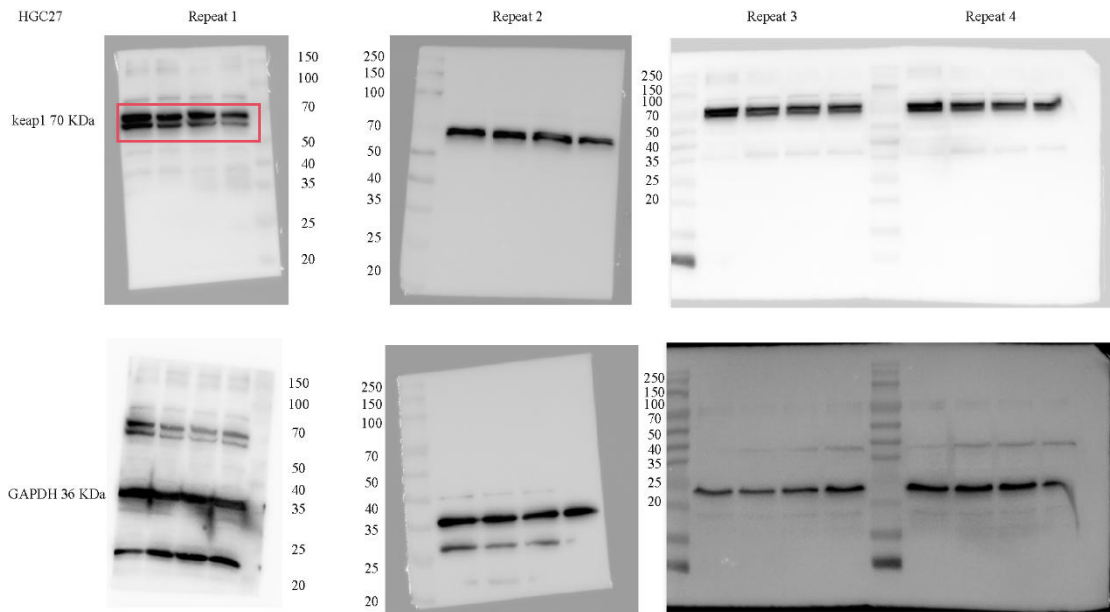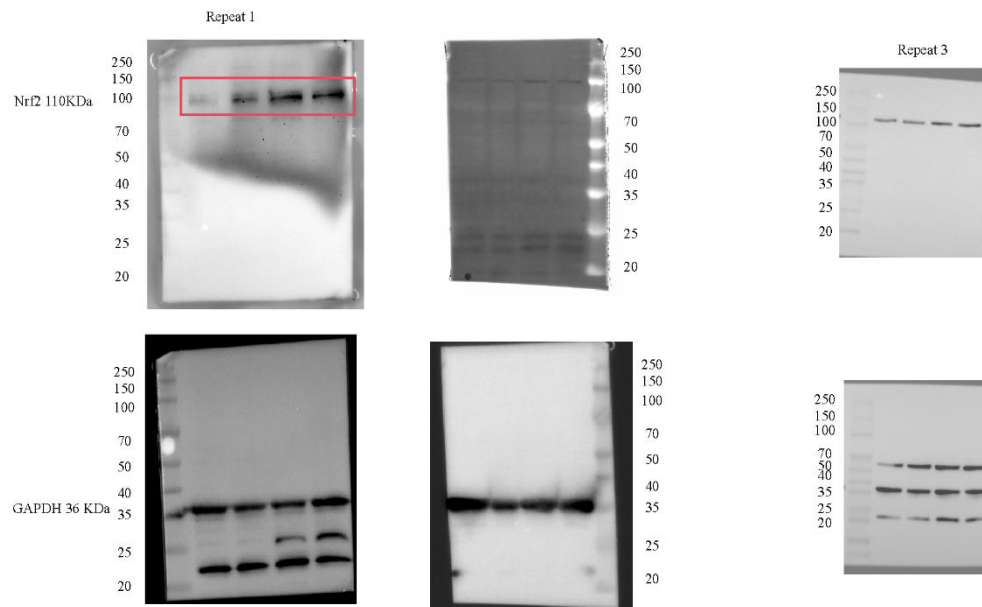

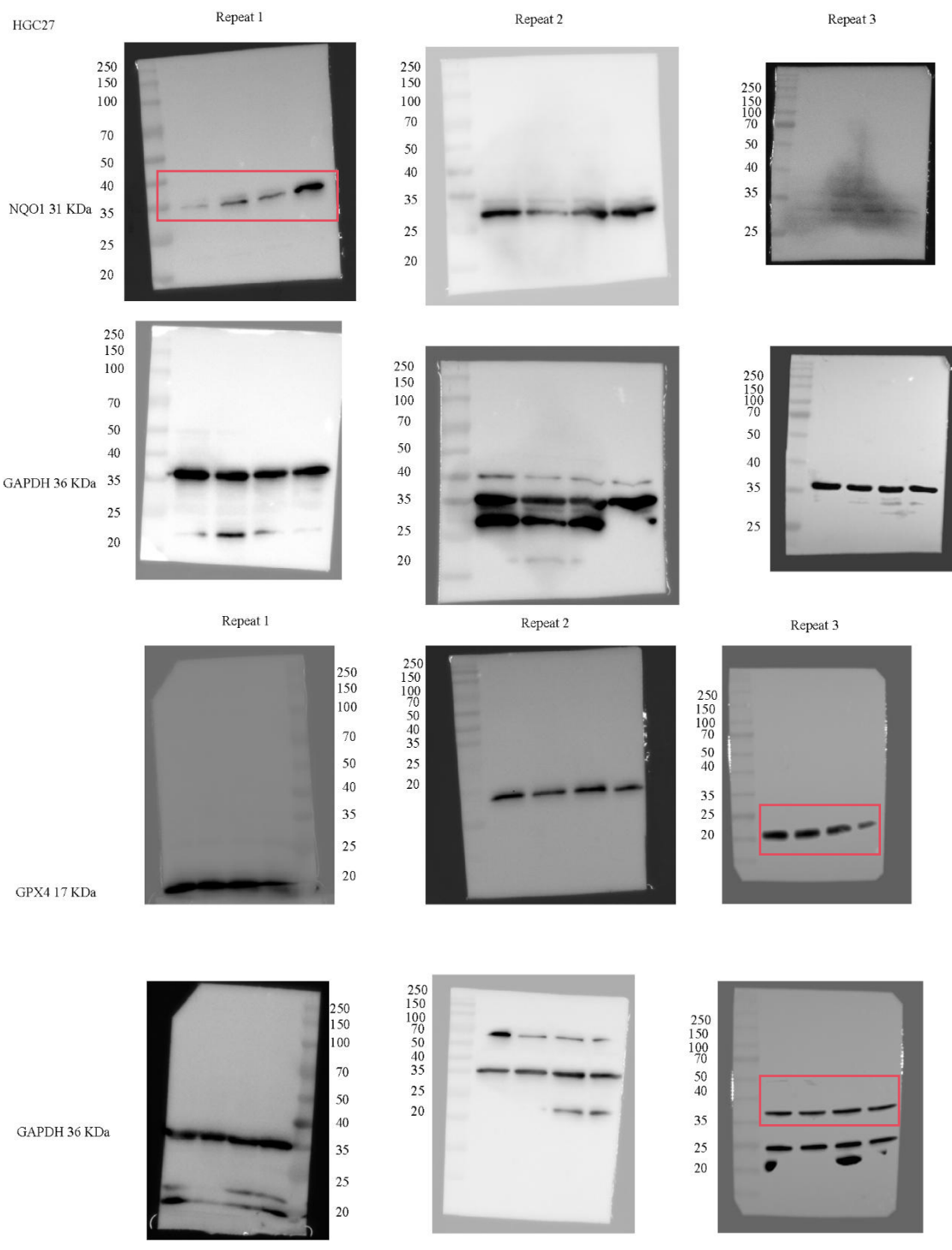

HGC27

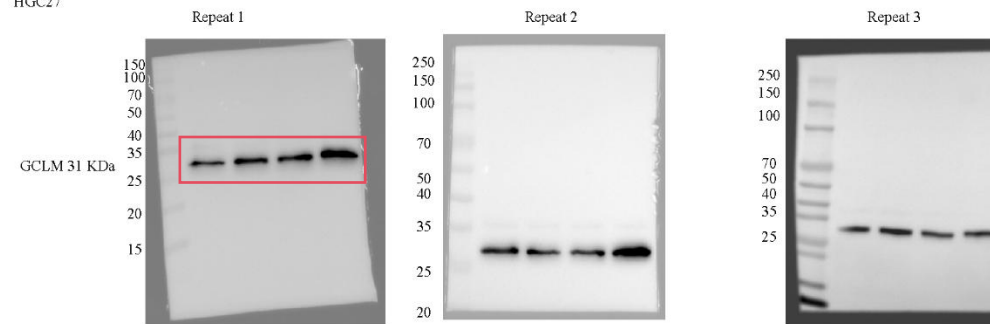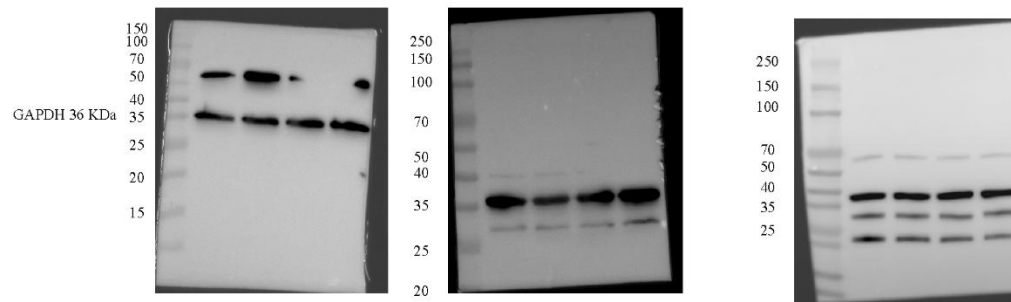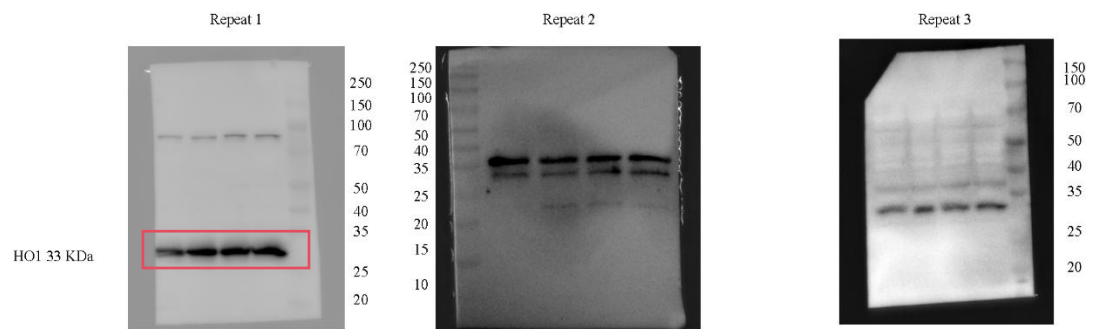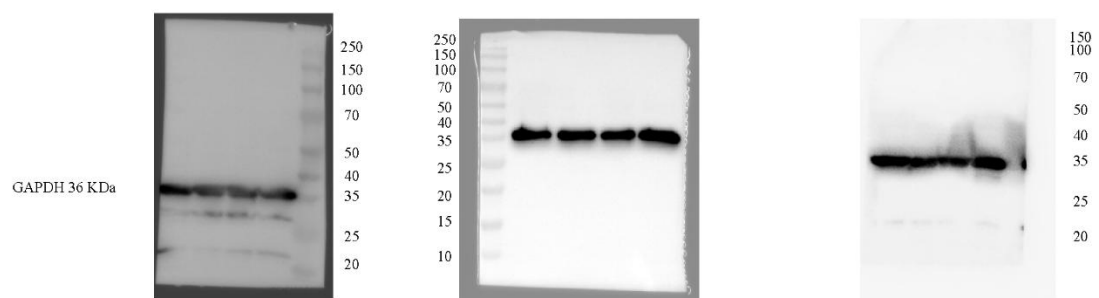

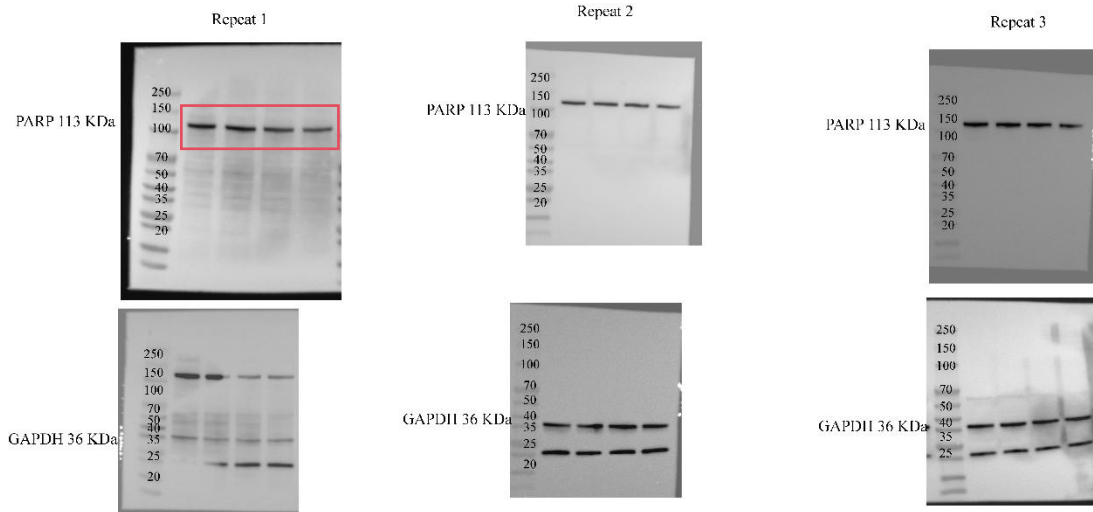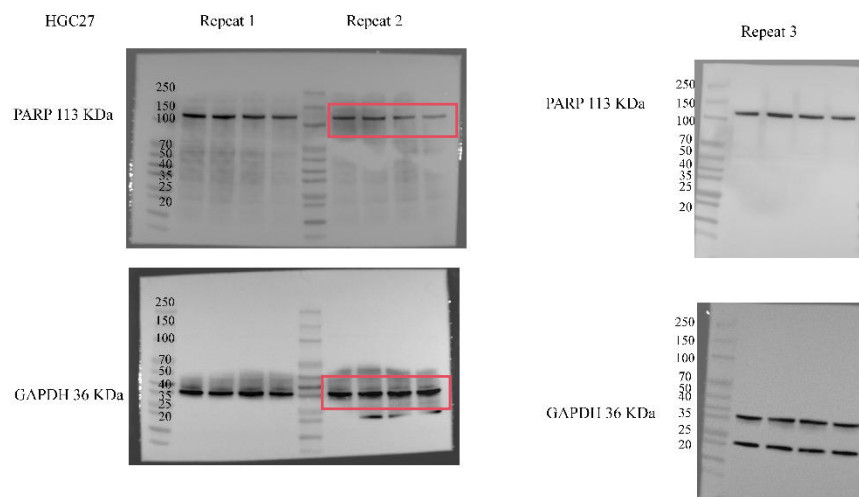

Supplement: Supplementary file 2 — Supplementary Information 2. [file 41598_2024_56431_MOESM2_ESM.pdf]
